# Lipid Paradox in Statin-Naïve Acute Ischemic Stroke But Not Hemorrhagic Stroke

**DOI:** 10.3389/fneur.2018.00541

**Published:** 2018-08-29

**Authors:** Kai-Hung Cheng, Jr-Rung Lin, Craig S. Anderson, Wen-Ter Lai, Tsong-Hai Lee, the SRICHS Group, Tsong-Hai Lee

**Affiliations:** Author Affiliations: Department of Neurology, Linkou Chang Gung Memorial Hospital, Taoyuan, Taiwan; Department of Neurology; Department of Emergency Medicine; Department of Medical Imaging and Intervention; Department of Neurology; Department of Emergency Medicine; Department of Medical Imaging and Intervention; Department of Neurology; Department of Emergency Medicine; Department of Medical Imaging and Intervention; Department of Neurology; Department of Emergency Medicine; Department of Medical Imaging and Intervention; ^1^Division of Cardiology, Department of Internal Medicine, Kaohsiung Medical University Hospital, Kaohsiung, Taiwan; ^2^Faculty of Medicine, College of Medicine, Kaohsiung Medical University, Kaohsiung, Taiwan; ^3^Clinical Informatics and Medical Statistics Research Center, Chang Gung University, Taoyuan, Taiwan; ^4^Neurological and Mental Health Division, The George Institute for Global Health, University of Sydney, Sydney, NSW, Australia; ^5^Neurology Department, Royal Prince Alfred Hospital, Sydney, NSW, Australia; ^6^Stroke Center and Department of Neurology, Linkou Chang Gung Memorial Hospital and College of Medicine, Chang Gung University, Taoyuan, Taiwan

**Keywords:** ischemic stroke, intracerebral hemorrhage, lipids, mortality, stroke outcome

## Abstract

**Background:** Low lipid level is associated with better cardiovascular outcome. However, lipid paradox indicating low lipid level having worse outcomes could be seen under acute injury in some diseases. The present study was designed to clarify the prognostic significance of acute-phase lipid levels within 1 day after admission for stroke on mortality in first-ever statin-naïve acute ischemic stroke (IS) and hemorrhagic stroke (HS).

**Methods:** This observational study was conducted using the data collected from Stroke Registry In Chang-Gung Healthcare System (SRICHS) between 2009 and 2012. Patients with recurrent stroke, onset of symptoms >1 day, and history of the use of lipid-lowering agents prior to index stroke were excluded. Stroke was classified into IS and hypertension-related HS. The primary outcomes were 30-day and 1-year mortality identified by linkage to national death registry for date and cause of death. Receiver operating characteristic (ROC) curve analysis and multivariate Cox proportional hazard models were used to examine the association of lipid profiles on admission with mortality.

**Results:** Among the 18,268 admitted stroke patients, 3,746 IS and 465 HS patients were eligible for analysis. In IS, total cholesterol (TC) <163.5 mg/dL, triglyceride (TG) <94.5 mg/dL, low-density lipoprotein (LDL) <100 mg/dL, non-high-density lipoprotein cholesterol (non-HDL-C) <130.5 mg/dL, and TC/HDL ratio <4.06 had significantly higher risk for 30-day/1-year mortality with hazard ratio (HR) of 2.05/1.37, 1.65/1.31, 1.68/1.38, 1.80/1.41, and 1.58/1.38, respectively, compared with high TC, TG, LDL, non-HDL-C, and TC/HDL ratio (*p* < 0.01 in all cases). In HS, lipid profiles were not associated with mortality, except HDL for 30-day mortality (*p* = 0.025) and high uric acid (UA) concentrations for 30-day and 1-year mortality (*p* = 0.002 and 0.012, respectively). High fasting glucose and high National Institute of Health Stroke Scale (NIHSS) score at admission were associated with higher 30-day and 1-year mortality in both IS and HS and low blood pressure only in IS (*p* < 0.05). Synergic effects on mortality were found when low lipids were incorporated with high fasting glucose, low blood pressure, and high NIHSS score in IS (*p* < 0.05).

**Conclusions:** Lipid paradox showing low acute-phase lipid levels with high mortality could be seen in statin-naïve acute IS but not in HS. The mortality in IS was increased when low lipids were incorporated with high fasting glucose, low blood pressure, and high NIHSS score.

## Introduction

The concept of “the lower the cholesterol, the better the outcome” is suggested for the prevention of cardiovascular events (1); however, there is inconsistent or weak association in the metabolic significance of lipids with stroke. Statin can lower cholesterol concentrations and help to reduce stroke risk in high-risk populations and in patients with non-cardioembolic stroke or transient ischemic attack (2). If statin therapy is discontinued between 3 and 6 months after an index ischemic stroke (IS), there is an increased risk of recurrent stroke within 1 year after statin discontinuation (3). High serum total cholesterol (TC) levels represent a risk factor of IS in Western countries, but it was found to be a risk factor mainly for large-artery occlusive infarction in Japanese men and not for lacunar or embolic infarction in either sex (4). The epidemiological studies in Eastern Asians have shown significantly inverse association between serum cholesterol and the risk of intracerebral hemorrhage (5–10). A recent community study in Japan found that high-density lipoprotein (HDL) levels had an inverse relationship with the incidence of lacunar infarction but a positive association with the risk of hemorrhagic stroke (HS), mainly in women (11). Lipid levels may be different between HS patients and non-HS controls, but a decline in serum TC and low-density lipoprotein (LDL) levels can be found within 6 months prior to primary HS, independent of statin treatment (12). These alterations in serum lipid trends may suggest a biological pathway to induce HS occurrence. However, the study of acute-phase lipid on stroke outcome is rare, and it is advised that further studies are needed to confirm the level of acute-phase lipid as a potential biomarker for brain injury.

The plasma concentration of LDL may increase with age, mainly as the result of reduced clearance of LDL and reduced conversion of cholesterol to bile acids with age (13), so it is likely that lipids may have more influence on the elderly than on young patients. Also, our previous study (14) has shown that the stroke etiology is different between young and elderly patients, and strokes of other determined etiology and undetermined etiology are the most common types among young stroke patients. As lipids may be involved in the progression of atherosclerosis, which is the most common stroke etiology in elderly patients, it is likely that lipids may play a more significant role in elderly stroke patients.

Reverse epidemiology or risk factor paradox has been mentioned in the case of body mass index, serum cholesterol, and blood pressure in elderly population (15, 16). However, there are limited outcome studies of acute-phase lipid in cerebrovascular and cardiovascular diseases. The present study intends to determine the association of acute-phase lipid levels within 1 day after admission for stroke, with short-term and long-term mortality in statin-naïve elderly Han-Chinese stroke patients with first-ever acute IS and HS.

## Materials and methods

### Study population and design

The clinical profiles of all patients with first-ever acute stroke prospectively registered in Stroke Registry In Chang-Gung Healthcare System (SRICHS) between 2009 and 2012 were retrieved and analyzed. The SRICHS was established in 2007, and the study was conducted in four branch hospitals of Chang-Gung Healthcare System, which includes two medical centers and two regional hospitals covering a population of 6.5 million from a total of 23 million people in Taiwan (17). All patients admitted with acute stroke received standard evaluations that included assessments of neurological severity using National Institutes of Health Stroke Scale (NIHSS) and disability on Modified Rankin Scale (MRS), as well as biochemical evaluations (including fasting blood glucose, uric acid (UA), and lipid profiles), monitoring of vital signs, and all imaging studies during their hospital stay.

Patients who presented with recurrent stroke (either by past history or as a recurrent event during study period), had a stroke with the onset of symptoms >1 day, and had a known or uncertain history of the use of lipid-lowering agents prior to index stroke were excluded. Patients aged ≤40 years were also excluded to prevent from recruiting patients with stoke caused by genetically related diseases such as familiar hypercholesterolemia, Moyamoya disease, or CADASIL. Stroke was classified into IS and HS. Ischemic stroke patients were recruited according to International Classification of Diagnosis (ICD-9-CM) codes 433-437, but excluded 430.00, 431.00, 433.20, 433.30, 433.80, 433.90, 434.90, 434.00, 434.10, and 434.90 “without mention of infarction”, 435 (transient cerebral ischemia) and 437.2–437.7 unless accompanied with a code for cerebral infarction or cerebral hemorrhage (18). Hemorrhagic stroke patients were recruited according to ICD-9-CM codes 430–432 and 432.1 but excluded secondary hemorrhages due to cerebral aneurysm, arteriovenous malformation/fistula, and cavernous hemangioma. Lobar and intraventricular hemorrhages were also excluded because of the high association with vascular anomaly in Asians (19, 20). The site of primary HS was defined as hypertension-related deep (i.e., thalamus, putamen, globus pallidus, or caudate nucleus) brainstem or cerebellum. The study was approved by the Institutional Review Board of Linkou Chang-Gung Memorial Hospital.

### Measures and outcomes

For baseline characteristics, smoking was recorded as the status of current smoking. The alcohol consumption was considered with ≥1 times per week within the past 6 months. Hypertension was identified either directly or from medical report, based on prior use of antihypertensive medication or in-hospital blood pressure (BP) >140/90 mmHg. Diabetes mellitus was identified either directly or from medical report, based on prior use of anti-diabetic medication or elevated in-hospital blood glucose (fasting ≥126 mg/dL, casual ≥200 mg/dL, or glycosylated hemoglobin ≥6.5%). The family history of stroke or coronary artery disease was also recorded. Fasting blood glucose, UA, and lipid profiles including TC, triglyceride (TG), LDL, HDL, non-HDL cholesterol (non-HDL-C), and TC/HDL ratio were routinely measured on the first working morning within 1 day of the stroke. All blood samples were examined at the Department of Laboratory Medicine using Hitachi LST008 (Japan).

According to the national policy, it is mandatory that all the death certificates issued by clinicians need to be reported to the Office of Statistics at the Ministry of Health and Welfare (MOHW) for registration in the national Taiwan death registry (TDR) (http://www.mohw.gov.tw/CHT/DOS/Statistic.aspx?f_list_no=474) (21). The ICD-9-CM codes are used to record the main cause of death on each death certificate (22). We linked the TDR data including the cause of death in ICD-9-CM codes and the date of death with the clinical information of SRICHS in Research Services Center for Health Information and Health and Welfare Data Science Center, Ministry of Health and Welfare-Chang Gung sub-center (http://rschi.cgu.edu.tw/bin/home.php?Lang=en). The Institutional Review Board of Chang Gung Memorial Hospital approved this linkage, and the privacy of the patients was protected.

### Statistical analysis

The primary outcomes of stroke were death at 30 days or death at 1 year. The first three leading causes of death were examined. Continuous variables were reported as median and interquartile range, and between-group comparisons were made by Mann-Whitney U test for continuous variables and chi-square test for categorical variables. The receiver operating characteristic (ROC) curve analysis was used to define the optimal cut-off points for each parameter for categorical data analysis from individual Youden's index for 30-day and 1-year mortality (Supplemental Figure 1). Multivariate Cox proportional hazard models were used to examine the effect of both categorical and continuous data of lipids on 30-day and 1-year mortality, and all lipid components were analyzed separately. Since categorical data were more convenient for clinical use to differentiate high-risk and low-risk patients and for the analysis of synergic effect when combined with other factors, we used categorical variables of lipids in our analysis. The linear regression was used to define the associations of stroke severity (NIHSS) with metabolic biomarkers and blood pressures. Statistical significance was set as a two-tailed test with *p*-value <0.05. The R (version 3.2.0) open source statistical software was used for analysis.

The interaction between age and sex was examined first, which showed significant interaction in IS (*p*-value for interaction = 0.032) but not in HS (*p*-value = 0.408). So, age and sex interaction was included for adjustment in the multivariate Cox proportional hazard model for IS but not for HS. The interaction between IS and HS was also examined (Supplemental Table 1). Since there was significant interaction between IS and HS in some lipids at 30-day mortality but in all at 1-year mortality, the IS and HS were analyzed separately.

## Results

### Baseline characteristics

**Figure 1:**
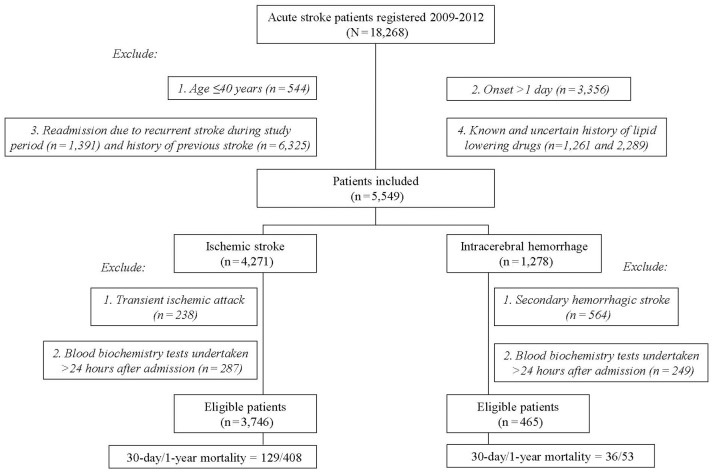
Flowchart of patient recruitment.

Among the 18,268 stroke patients in the SRICHS database for the study period, we excluded those with recurrent stroke (*n* = 7,716), age ≤ 40 years (544), stroke onset >1 day (3,356), known/uncertain history of the use of lipid-lowering agents (3,550), transient ischemic attack coded as IS (238), secondary HS (564), and blood biochemistry tests undertaken 24 h after admission (IS = 287, HS = 249). Thus, 3,746 IS and 465 primary HS patients were eligible for analysis (Figure 1), and their profiles are outlined in Table 1. Since patients with hypertension, no diabetes, or no atrial fibrillation had higher frequency of low lipid levels (Supplemental Table 2), these risk factors were included for adjustment in multivariate Cox proportional hazard model. Table 1 shows that old age and history of atrial fibrillation were significantly associated with high mortality, whereas male gender, smoking, alcohol drinking, and a family history of stroke were associated with low mortality in patients with statin-naïve IS. However, these factors had no significant difference between the survivors and the deaths in statin-naïve HS.

**Table 1:** Clinical profiles of 30-day and 1-year mortality in first-ever statin-naïve acute ischemic and intracerebral hemorrhagic stroke.

	**Ischemic stroke**						**Hemorrhagic stroke**					
	**30-day**			**1-year**			**30-day**			**1-year**		
	**Survival**	**Mortality**		**Survival**	**Mortality**		**Survival**	**Mortality**		**Survival**	**Mortality**	
**Clinical profile**	***n* = 3,617**	***n* = 129**	***p*-value**	***n* = 3,338**	***n* = 408**	***p*-value**	***n* = 429**	***n* = 36**	***p*-value**	***n* = 412**	***n* = 53**	***p*-value**
Age, years	69 (59–78)	78 (70–86)	< 0.001^*^	68 (58–77)	79 (71–86)	< 0.001^*^	60 (53,71)	60 (53,75)	0.810	59 (53,70)	62 (53,77)	0.056
Male	2,194 (61)	61 (47)	0.002^*^	2,035 (61)	220 (54)	0.006^*^	263 (61.3)	25 (69)	0.334	252 (61)	36 (68)	0.340
**MEDICAL HISTORY**												
Hypertension	2,660 (74)	92 (71)	0.574	2,462 (74)	290 (71)	0.247	368 (86)	29 (81)	0.394	353 (86)	44 (83.0)	0.606
Diabetes	1,210 (34)	41 (32)	0.693	1,100 (33)	151 (37)	0.101	87 (20)	12 (33)	0.066	83 (20)	16 (30)	0.093
AF	675 (19)	55 (43)	< 0.001^*^	570 (17)	160 (39)	< 0.001^*^	30 (7)	–^a^	0.743	27 (7)	5 (9)	0.623
Smoking	1,169 (32)	28 (22)	0.011^*^	1,103 (33)	94 (23)	< 0.001^*^	176 (41)	18 (50.0)	0.294	171 (42)	23 (43)	0.793
Alcohol	587 (16)	11 (9)	0.019^*^	558 (17)	40 (10)	< 0.001^*^	152 (35.4)	9 (25)	0.206	147 (36)	14 (26)	0.182
**FAMILY HISTORY**												
Stroke	346 (10)	5 (4)	0.029^*^	337 (10)	14 (3)	< 0.001^*^	37 (9)	–^a^	0.219	36 (9)	–^a^	0.214
CAD	99 (3)	0 (0)	0.104	95 (3)	4 (1)	0.027^*^	13 (3)	–	0.594	13 (3.2)	–	0.385

* *p < 0.05. CAD denotes coronary artery disease; AF, atrial fibrillation*.

a *indicates the number of patient ≤ 2*.

*Median (interquartile range) for continuous variables using Mann-Whitney U test; numbers (%) for categorical variables using chi-square test*.

### Receiver operating characteristic (roc) curves (Supplemental Figure 1)

In IS, the cut-off points for multivariate Cox proportional hazard model were obtained from ROC curves except for LDL, systolic blood pressure, and UA which were defined from normal levels according to local guideline. In HS, the cut-off points for diastolic and mean blood pressure, fasting glucose, and NIHSS score were obtained from ROC curves, and the rest were obtained from normal levels according to local guideline (Supplemental Figure 1).

### Mortality and lipid variables in acute ischemic stroke (Table 2) and hemorrhagic stroke (Table 3)

In IS, TC < 163.5 mg/dL, TG < 94.5 mg/dL, LDL < 100 mg/dL, non-HDL-C < 130.5 mg/dL, and TC/HDL ratio < 4.06 had significantly higher risk for 30-day mortality with hazard ratio (HR) of 2.05 (*p* < 0.001), 1.65 (*p* = 0.005), 1.68 (*p* = 0.002), 1.80 (*p* = 0.001), and 1.58 (*p* = 0.009), respectively, compared with high TC, TG, LDL, non-HDL-C, and TC/HDL ratio. Also, low TC, TG, LDL, non-HDL-C, and TC/HDL ratio had significantly higher risk for 1-year mortality with HR of 1.37, 1.31, 1.38, 1.41, and 1.38, respectively (*p* < 0.01 in all cases). The results using continuous variables were presented in Supplemental Table 3-1, which showed that only TC, LDL, and non-HDL-C had significance at 1-year. In HS, there was no significant association between lipids and mortality at both time points, except HDL for 30-day mortality (HR = 0.44, *p* = 0.025). The results using continuous variables were presented in Supplemental Table 3-2, which showed that none of the lipids had significance at 30-day and 1-year (Tables 2, 3).

**Table 2:** Clinical findings and hazard ratio of 30-day and 1-year mortality in first-ever statin-naïve acute ischemic stroke.

**Clinical findings and NIHSS**	**Ischemic stroke–30-day**								**Ischemic stroke–1-year**							
	**Survival**	**Mortality**		**Cut-off point**	**HR**	**95%CI**			**Survival**	**Mortality**		**Cut-off point**	**HR**	**95% CI**		
	***n* = 3,617**	***n* = 129**	***p*-value**			**Lower**	**Upper**	***p*-value**	***n* = 3,338**	***n* = 408**	***p*-value**			**Lower**	**Upper**	***p*-value**
Total cholesterol (mg/dL)	179 (154, 206)	160 (133, 198)	< 0.001^*^	163.5	2.05	1.47	2.86	< 0.001^*^	180 (155, 207)	166 (136, 195)	< 0.001^*^	163.5	1.37	1.13	1.66	0.001^*^
Triglyceride (mg/dL)	108 (78, 155)	88 (59, 117)	< 0.001^*^	94.5	1.65	1.16	2.34	0.005^*^	110 (79, 157)	89 (66, 119)	< 0.001^*^	94.5	1.31	1.08	1.60	0.007^*^
Low-density lipoprotein (LDL; mg/dL)	111 (90, 134)	100 (74, 129)	0.004^*^	100	1.68	1.21	2.33	0.002^*^	112 (91, 135)	101 (79, 126)	< 0.001^*^	100	1.38	1.15	1.67	0.001^*^
High-density lipoprotein (HDL; mg/dL)	41 (35, 50)	43 (33, 53)	0.835	39.8	1.37	0.99	1.89	0.059	41 (35, 50)	42 (33, 52)	0.914	43.2	1.00	0.83	1.22	0.973
Non-HDL cholesterol (mg/dL)	135 (111, 162)	116 (91, 150)	< 0.001^*^	130.5	1.80	1.27	2.57	0.001^*^	136 (113, 162)	119 (94, 149)	< 0.001^*^	130.5	1.41	1.16	1.72	0.001^*^
Total cholesterol/HDL ratio	4.3 (3.4,5.2)	3.8 (3.2,5.0)	0.009^*^	4.06	1.58	1.13	2.23	0.009^*^	4.3 (3.5,5.2)	3.8 (3.1,4.9)	< 0.001^*^	4.04	1.38	1.13	1.67	0.001^*^
Systolic blood pressure (mmHg)	151 (134, 170)	147 (128, 174)	0.164	140	1.37	0.99	1.90	0.058	151 (135,170)	148 (130,170)	0.009^*^	140	1.30	1.08	1.58	0.006^*^
Diastolic blood pressure (mmHg)	85 (76, 96)	79 (67, 95)	< 0.001^*^	79.5	1.82	1.32	2.51	< 0.001^*^	86 (77, 96)	80 (70,93)	< 0.001^*^	69.5	1.80	1.44	2.24	< 0.001^*^
Mean blood pressure (mmHg)	108 (97, 120)	103 (88, 119)	0.008^*^	99.8	1.80	1.31	2.48	< 0.001^*^	108 (97, 121)	103 (91, 117)	< 0.001^*^	99.8	1.40	1.16	1.69	< 0.001^*^
Fasting glucose (mg/dL)	114 (95, 152)	148 (113, 190)	< 0.001^*^	108.9	3.44	2.41	4.92	< 0.001*	114 (94, 151)	133 (106, 181)	< 0.001^*^	108.9	1.90	1.57	2.31	< 0.001^*^
Uric acid (mg/dL)	5.5 (4.5, 6.6)	5.4 (4.0, 7.0)	0.608	8.0	0.87	0.46	1.66	0.676	5.5 (4.5,6.6)	5.7 (4.1,7.0)	0.890	8.0	1.13	0.81	1.56	0.480
NIHSS score	4.0 (2.0, 9.0)	20 (14, 29)	< 0.001^*^	12.5	17.1	11.2	26.1	< 0.001^*^	4.0 (2.0,8.0)	14 (6.0,22)	< 0.001^*^	10.5	4.81	3.92	5.90	< 0.001^*^

* *p < 0.05. NIHSS, National Institute of Health Information; HR, hazard ratio; CI, confidence interval. Median (Q1,Q3) are reported for continuous variables using Mann-Whitney U test*.

*Multivariate Cox proportional hazard model for dichotomous data after adjusting age, sex, age × sex (interaction), atrial fibrillation, smoking, alcohol, hypertension, diabetes mellitus, and family history of coronary artery disease for 30-day mortality; age, sex, atrial fibrillation, smoking, alcohol, hypertension, diabetes mellitus, family history of coronary artery disease and family history of stroke for 1-year mortality*.

*Cut-off points are obtained from receiver operating characteristic curves except low-density lipoprotein, systolic blood pressure and uric acid which are obtained from normal levels according to local guideline*.

**Table 3:** Clinical findings and hazard ratio of 30-day and 1-year mortality in first-ever statin-naïve acute intracerebral hemorrhagic stroke.

**Clinical findings and NIHSS**	**Hemorrhagic stroke- 30-day**								**Hemorrhagic stroke- 1-year**							
	**Survival**	**Mortality**		**Cut-off point**	**HR**	**95% CI**			**Survival**	**Mortality**		**Cut-off point**	**HR**	**95% CI**		
	***n* = 429**	***n* = 36**	***p*-value**			**Lower**	**Upper**	***p*-value**	***n* = 412**	***n* = 53**	***p*-value**			**Lower**	**Upper**	***p*-value**
Total cholesterol (TC; mg/dL)	180 (153, 206)	168 (139, 208)	0.276	200	0.88	0.43	1.78	0.72	180 (154, 206)	167 (143, 208)	0.196	200	0.96	0.53	1.75	0.895
Triglyceride (TG; mg/dL)	106 (79, 148)	103 (79, 174)	0.942	150	0.82	0.39	1.75	0.614	107 (80,148)	97 (75,143)	0.363	150	0.85	0.44	1.64	0.631
Low-density lipoprotein (LDL; mg/dL)	110 (86, 132)	101 (74, 141)	0.573	100	1.18	0.59	2.34	0.642	110 (86, 132)	101 (75, 138)	0.572	100	1.12	0.64	1.98	0.689
High-density lipoprotein (HDL; mg/dL)	45 (37, 55)	48 (40, 52)	0.816	45	0.44	0.21	0.90	0.025^*^	45 (37, 55)	47 (37, 53)	0.766	45	0.61	0.35	1.07	0.085
Non-HDL cholesterol (N-HDL-C; mg/dL)	130 (106, 160)	124 (96, 162)	0.453	130	0.92	0.46	1.82	0.807	130 (106, 160)	126 (96, 162)	0.395	130	0.95	0.55	1.67	0.868
Total cholesterol/HDL ratio (TC/HDL)	3.9 (3.1, 4.9)	3.8 (3.1, 4.9)	0.968	5.0	0.85	0.39	1.84	0.677	3.9 (3.1, 4.9)	3.9 (3.1, 4.9)	0.908	5.0	0.78	0.41	1.48	0.446
Systolic blood pressure (SBP; mmHg)	157 (142, 171)	161 (129, 195)	0.582	140	1.75	0.86	3.57	0.121	156 (141, 171)	158 (137, 188)	0.521	140	1.37	0.74	2.52	0.317
Diastolic blood pressure (DBP; mmHg)	89 (80, 100)	86 (71, 106)	0.584	77.5	1.91	0.94	3.90	0.075	90 (80,100)	85 (72,105)	0.260	78.5	1.72	0.95	3.11	0.074
Mean blood pressure (MBP; mmHg)	112 (101, 124)	115 (93, 134)	0.918	106.7	1.29	0.66	2.55	0.458	112 (102,124)	109 (95,132)	0.817	106.7	1.27	0.73	2.23	0.395
Fasting glucose (FG; mg/dL)	109 (96, 137)	163 (141, 238)	<0.001^*^	128.5	14.3	3.04	67.5	0.001^*^	108 (96, 136)	149 (130, 236)	<0.001^*^	128.5	7.11	2.54	19.9	<0.001^*^
Uric acid (UA; mg/dL)	5.5 (4.4, 6.8)	6.7 (5.7, 9.6)	0.005^*^	8.0	4.61	1.75	12.2	0.002^*^	5.5 (4.4, 6.8)	6.1 (5.0, 7.9)	0.027^*^	8.0	3.18	1.29	7.83	0.012^*^
NIHSS score	8.0 (4.0,14)	38 (38,40)	<0.001^*^	21.5	111	26.5	463	<0.001^*^	7.5 (3.0,14)	38 (20,40)	<0.001^*^	18.5	20.3	10.4	39.7	<0.001^*^

* *p < 0.05. NIHSS, National Institute of Health Information; HR, hazard ratio; CI, confidence interval. Median (Q1,Q3) are reported for continuous variables using Mann-Whitney U test*.

*Multivariate Cox proportional hazard model for dichotomous data after adjusting age, sex, hypertension, and diabetes mellitus for 30-day and 1-year mortality in hemorrhagic stroke*.

*Cut-off points for diastolic blood pressure, fasting glucose, and NIHSS score are obtained from receiver operating characteristic curves, and the rest are obtained from normal levels according to local guideline*.

### Synergic effects of lipid with mortality in acute ischemic stroke

For 30-day mortality, low TC, TG, LDL, non-HDL-C, and TC/HDL ratio significantly increased the HR of high NIHSS from 17.1 to 43.9, 21.5, 38.2, 35.5, and 21.0; increased the low mean BP from 1.80 to 3.18, 2.83, 2.74, 2.64, and 2.52; and also increased the high fasting glucose from 3.44 to 8.08, 6.10, 6.66, 6.89, and 5.51, respectively (Tables 2, 4, all *p* < 0.01). For 1-year mortality, low TC, TG, LDL, non-HDL-C, and TC/HDL ratio significantly increased the HR of high NIHSS from 4.81 to 6.61, 5.45, 6.83, 7.11, and 5.97; increased the low mean BP from 1.40 to 1.89, 1.73, 1.92, 1.90, and 1.87; and also increased the high fasting glucose from 1.90 to 2.93, 2.65, 2.85, 2.94, and 2.78, respectively (Tables 2, 4, all *p* < 0.01).

**Table 4:** Hazard ratio of 30-day and 1-year mortality according to lipid levels with NIHSS score, DBP and fasting glucose in first-ever statin-naïve acute ischemic stroke.

	**30-day**				**1-year**			
	**HR**	**95% CI**		***P*-value**	**HR**	**95% CI**		***P*-value**
		**Lower**	**Upper**			**Lower**	**Upper**	
**VARIABLES AT ADMISSION IN ISCHEMIC STROKE**								
**Low TC**	2.05	1.47	2.86	<0.001^*^	1.37	1.13	1.66	0.001^*^
Low TC and high NIHSS	43.9	22.1	87.4	<0.001^*^	6.61	4.91	8.88	<0.001^*^
Low TC and low MBP	3.18	2.10	4.80	0.001^*^	1.89	1.47	2.44	<0.001^*^
Low TC and high FG	8.08	4.67	14.0	<0.001^*^	2.93	2.21	3.87	<0.001^*^
**Low TG**	1.65	1.16	2.34	0.005^*^	1.31	1.08	1.60	0.007^*^
Low TG and high NIHSS	21.5	11.5	40.3	<0.001^*^	5.45	4.05	7.33	<0.001^*^
Low TG and low MBP	2.83	1.76	4.57	<0.001^*^	1.73	1.31	2.30	<0.001^*^
Low TG and high FG	6.10	3.45	10.8	<0.001^*^	2.65	1.98	3.54	<0.001^*^
**Low LDL**	1.68	1.21	2.33	0.002	1.38	1.15	1.67	<0.001^*^
Low LDL and high NIHSS	38.2	19.2	76.3	<0.001^*^	6.83	5.07	9.21	<0.001^*^
Low LDL and low MBP	2.74	1.82	4.13	<0.001^*^	1.92	1.49	2.47	<0.001^*^
Low LDL and high FG	6.66	3.85	11.5	<0.001^*^	2.85	2.15	3.78	<0.001^*^
**Low Non-HDL-C**	1.80	1.27	2.57	0.001^*^	1.41	1.16	1.72	0.001^*^
Low Non-HDL-C and high NIHSS	35.5	17.4	72.5	<0.001^*^	7.11	5.20	9.72	<0.001^*^
Low Non-HDL-C and low MBP	2.64	1.72	4.06	<0.001^*^	1.90	1.45	2.48	<0.001^*^
Low Non-HDL-C and high FG	6.89	3.80	12.5	<0.001^*^	2.94	2.19	3.95	<0.001^*^
**Low TC/HDL ratio**	1.58	1.13	2.23	0.009^*^	1.38	1.13	1.67	0.001^*^
Low TC/HDL ratio and high NIHSS	21.0	11.6	38.1	<0.001^*^	5.97	4.44	8.02	<0.001^*^
Low TC/HDL and low MBP	2.52	1.61	3.96	<0.001^*^	1.87	1.41	2.46	<0.001^*^
Low TC/HDL and high FG	5.51	3.17	9.58	<0.001^*^	2.78	2.09	3.69	<0.001^*^

* *p < 0.05. DBP, diastolic blood pressure; HR, hazard ratio; CI, confidence interval; NIHSS, National Institute of Health Information; TC, total cholesterol; TG, triglyceride; FG, fasting glucose; LDL, low-density lipoprotein; HDL, high-density lipoprotein; Non-HDL-C, Non-HDL cholesterol; TC/HDL, total cholesterol/HDL ratio; UA, uric acid. Cut-off points of TC, NIHSS, SBP, FG, LDL, Non-HDL-C and TC/HDL = 163.5/163.5 mg/dL, 12.5/10.5, 140/140 mmHg, 108.9/108.9 mg/dL, 100/100 mg/dL, 130.5/130.5 mg/dL and 4.06/4.04 for 30-day/1-year mortality in first-ever ischemic stroke. Multivariate Cox proportional hazard model after adjusting age, sex, age × sex (interaction), atrial fibrillation, smoking, alcohol, hypertension, diabetes mellitus, and family history of stroke for 30-day mortality; age, sex, age × sex (interaction), atrial fibrillation, smoking, alcohol, hypertension, diabetes mellitus, family history of coronary artery disease and family history of stroke for 1-year mortality in first-ever ischemic stroke*.

### Mortality and other metabolic and hemodynamic variables in ischemic and hemorrhagic stroke

High fasting glucose significantly increased the HR of 30-day and 1-year mortality in both IS (HR = 3.44 and 1.90, respectively) and HS (HR = 14.3 and 7.11, respectively) (*p* < 0.001 in all cases). Low diastolic BP and mean BP significantly increased the HR by 1.82 and 1.80 for 30-day mortality and by 1.80 and 1.40 for 1-year mortality (*p* < 0.001 in all cases) in IS but not in HS (Tables 2, 3). There were no significant correlations of TC, TG, LDL, non-HDL-C, and TC/HDL ratio with fasting glucose and UA, as well as fasting glucose with UA (Supplemental Table 4a–d). High UA was found to have significantly higher risk for 30-day and 1-year mortality (HR = 4.61 and 3.18, *p* = 0.002 and 0.012, respectively) in HS but not in IS.

### Admission stroke severity (NIHSS) and hemodynamic and metabolic variables

The NIHSS score showed a significantly negative association with TC, TG, non-HDL-C, TC/HDL ratio, and BP (diastolic and mean) but a positive association with HDL and fasting glucose in IS on linear regression analysis. There was significantly negative association of NIHSS score with diastolic BP and positive association with fasting glucose in HS (Table 5).

**Table 5:** Relationship between NIHSS score and clinical and laboratory findings in first-ever statin-naïve acute ischemic and intracerebral hemorrhagic stroke.

	**Ischemic stroke**				**Hemorrhagic stroke**			
	**β**	**95% CI**		***p*-value**	**β**	**95% CI**		***p*-value**
		**Lower**	**Upper**			**Lower**	**Upper**	
Total cholesterol (mg/dL)	−0.01	−0.01	0.00	0.010^*^	−0.02	−0.05	0.00	0.061
Triglyceride (mg/dL)	−0.01	−0.01	−0.01	<0.001^*^	0.00	−0.01	0.01	0.781
Low-density lipoprotein (mg/dL)	−0.01	−0.01	0.00	0.147	−0.02	−0.05	0.01	0.143
High-density lipoprotein (HDL; mg/dL)	0.03	0.01	0.05	0.003^*^	−0.07	−0.14	0.01	0.081
Non-HDL cholesterol (mg/dL)	−0.01	−0.02	0.00	<0.001^*^	−0.02	−0.04	0.01	0.232
Total cholesterol/HDL ratio	−0.17	−0.32	−0.02	0.024^*^	0.27	−0.21	0.75	0.274
Systolic blood pressure (mmHg)	−0.01	−0.02	0.00	0.155	−0.01	−0.04	0.03	0.777
Diastolic blood pressure (mmHg)	−0.04	−0.05	−0.02	<0.001^*^	−0.08	−0.14	−0.02	0.006^*^
Mean blood pressure (mmHg)	−0.02	−0.04	−0.01	0.001^*^	−0.05	−0.11	0.00	0.068
Fasting glucose (mg/dL)	0.02	0.02	0.03	<0.001^*^	0.06	0.04	0.08	<0.001^*^
Uric acid (mg/dL)	0.04	−0.12	0.19	0.643	0.34	−0.27	0.95	0.277

* *p < 0.05. NIHSS, National Institutes of Health Stroke Scale*.

*Linear regression without adjusting confounding factors*.

### Cause of death

Recurrent stroke was the leading cause of death in both IS and HS at 30 days (57 and 84%) and 1 year (35 and 73%). Other major causes of death in IS were cardiac disease (14%) and concomitant cancer (8%) at 30 days and concomitant cancer (15%) and cardiac disease (12%) at 1 year. Diabetes was the second leading cause of death in HS at both 30 days and 1 year (4 and 5%, respectively; Table 6).

**Table 6:** The first three leading causes of death at 30 days and 1 year in different stroke subtype.

**A. 30 days**			
Ischemic stroke mortality, *n* = 153		Hemorrhagic stroke mortality, *n* = 121	
Cause of death	*n* (%)	Cause of death	*n* (%)
**1. Cerebrovascular disease**	87 (56.9)	**1. Cerebrovascular disease**	102 (84.3)
Ischemic stroke	82	Hemorrhagic stroke	98
Hemorrhagic stroke	5	Ischemic stroke	4
**2. Heart disease**	22 (14.4)	**2. Diabetes mellitus**	5 (4.1)
Acute myocardial infarction	6		
Cardiac arrhythmia	6		
Others	10		
**3. Concomitant cancer**	12 (7.8)	**3. Concomitant cancer**	<= 2
Lung cancer	6		
Others	6		
**B. 1 year**			
Ischemic stroke mortality, *n* = 452		Hemorrhagic stroke mortality, *n* = 165	
Cause of death	*n* (%)	Cause of death	*n* (%)
**1. Cerebrovascular disease**	157 (34.7)	**1. Cerebrovascular disease**	121 (73.3)
Ischemic stroke	145	Hemorrhagic stroke	108
Hemorrhagic stroke	12	Ischemic stroke	13
**2. Concomitant cancer**	67 (14.8)	**2. Diabetes mellitus**	8 (4.9)
Lung cancer	22		
Liver cancer	11		
Others	34		
**3. Heart disease**	53 (11.7)	**3. Hypertension**	6 (3.6)
Acute myocardial infarction	20		
Cardiac arrhythmia	8		
Others	25		

## Discussion

This large prospective multisite registry study is the first study to investigate the associations of acute-phase lipid levels within 1 day after admission for stroke in both statin-naïve acute IS and HS. Our results demonstrated that low TC, TG, LDL, non-HDL-C, and TC/HDL ratio could be predictive of both 30-day and 1-year mortality in IS but not in HS. Compared with other clinical and radiological measures, NIHSS is regarded as the most sensitive outcome measure for stroke study (23, 24), and it is usually recommended to be considered in stroke outcome studies. Our results showed that the predictive value of low lipids was independent of admission NIHSS score and also of fasting glucose and BP profiles, and there was a synergistic effect on mortality after incorporating lipids with each of them (Table 4).

### Acute-phase lipid as a predictor for mortality in acute ischemic stroke

Cholesterol is an essential element of cell membranes, intracellular transport, and cell signaling (25), and lowered cholesterol levels may alter the membrane fluidity by increasing the membrane packing of phospholipid fatty-acids and the membrane permeability to neutral solutes (26) and hydrogen and sodium ions (27), resulting in the failure of neuronal cells' ability to resist local hyperosmolarity and acidosis under ischemic stress. In addition, cholesterol is a precursor of stress hormones, such as cortisol, which help to cope with life-threatening stress. So, lowered cholesterol could be harmful under acute injury.

High admission cholesterol was reported to be associated with good long-term survival after acute IS (28), and low serum cholesterol was associated with increased stroke severity and poor functional outcome in acute IS patients with or without prestroke statin treatment (29). However, these studies were complicated by some limitations including small samples, uncertain onset of symptoms, measurement error, uncertain statin effect due to prior statin use, and an inability to adjust for other biochemical indices such as glucose and cholesterol subfractions (28, 29). Our study was conducted in a large population with statin-naïve stroke patients to eliminate the influence of statin on stroke outcome and with detailed cholesterol subfractions to examine the effect of different lipids. Also, stroke severity, fasting glucose, and BP data were included for risk assessments.

Lipid paradox may be associated with the risk of mortality. Observational cohort studies have revealed that people with low TC levels (e.g., TC <154.4 mg/dL) have increased risk of subsequent death in some cancers, respiratory diseases, digestive diseases, trauma, and other non-medical causes than people with high baseline cholesterol levels (30, 31). This lipid paradox was also found to be associated with increased risk of death in acute myocardial infarction (32), elderly people (33, 34), and heart failure (33, 34). Lipid paradox may also be associated with the risk of disease. High TC and LDL was reported to be associated with a lower risk of atrial fibrillation (35), and lipids may have paradoxical associations with the risk of cardiovascular disease in rheumatoid arthritis (36). In the study of cerebrovascular disease, low TC levels may increase the risk of HS (5–12) and also increase the risk of hemorrhagic transformation after IS (37, 38). However, low TC was reported to affect the outcome by increasing the risk of mortality in IS (28, 29).

A recent study in acute HS demonstrated that TC level <160 mg/dL was associated with increased neurological severity and worse 3-month outcomes (39). Unlike the previous study, which discussed all the causes of HS, our study focused on the first-ever statin-naïve acute HS with stroke onset <1 day that was specifically related to hypertension but not to other causes such as vascular anomaly. Our study found that 5 out of 87 stroke mortalities (5.7%) were due to HS at 30 days and 12 out of 157 stroke mortalities (7.6%) were due to HS at 1 year (Table 6). However, our study failed to determine the frequency of HS occurrence under low TC, and further investigation is needed for the same.

Non-HDL-C is suggested to be a more reliable target for the treatment of dyslipidemia than LDL in patients with acute myocardial infarction (40), but it is rarely discussed in acute stroke. Epidemiological studies have demonstrated inconclusive results in the general Japanese population, indicating that non-HDL-C levels could have association with long-term mortality caused by stroke (41) or not by stroke (42). Our study found that similar to TC, admission non-HDL-C level is predictive of both 30-day and 1-year mortality in IS but not in HS.

The association of HDL with stroke severity (NIHSS) and stroke outcome in IS was controversial, with no association in some (43–45) and good association in others (46, 47). However, the TC/HDL (44, 45) and TG/HDL ratio (44) showed good association with stroke severity and stroke outcome in IS. Our results showed that high acute-phase HDL had association with 30-day mortality in HS (Table 3), and with stroke severity in IS (Table 5), and low TC/HDL ratio had association with both 30-day and 1-year mortality in IS. Although acute stroke relative to controls may have an increased proportion of dysfunctional HDL with abundant myeloperoxidase and α1-antitrypsin, resulting in an inappropriate protection of endothelial cells under stressful conditions (48), the association between HDL and stroke outcome is still inconclusive.

Previous studies have showed that low TG concentration predicts high mortality after stroke (49, 50), and it is associated with more severe stroke (51). Serum LDL, TG, and TC levels were significantly lower in ischemic patients with hemorrhagic transformation (37, 38). Our previous study in acute myocardial infarction found that acute-phase low TG and low LDL were associated with significantly higher 30-day in-hospital mortality (32). Our present results also found an association of low TG and low LDL with high 30-day and 1-year mortality in IS but not in HS.

It is reported that in the early phase after acute IS onset, there is a remarkable change in the level of serum lipids. Only the decreased level of HDL at 48 h after stroke onset and at discharge and the increased levels of TC and LDL at 24 h, 48 h, and at discharge were reported to be associated with poor functional outcome (46). Serum TC level may decrease soon after an acute stroke (52–56) and then increase to the highest level at 3 months. The possible explanation for the discrepancy in the association between lipids and outcome in different stages of stroke could be that low lipid in acute phase may be harmful to ischemic cells that are under acute injury, but low lipid in stationary phase could help to prevent the progression of atherosclerosis and also prevent the occurrence of vascular events.

### Acute-phase uric acid, glucose, and blood pressure in acute IS and HS

The levels of UA were reported to be positively associated with stroke (57). A previous study (58) showed that in overall stroke, elevated serum UA was associated with an increased risk of early death, but in acute HS, the association was seen only in univariate analysis but not in multivariate analysis. Our result revealed that the association of UA was seen only in HS but not in IS. However, we did not examine the history of previous medications for lowering UA, so our results are likely to be fragile. Further studies may clarify the association.

Acute-phase glucose level showed a significant correlation with mortality and morbidity in acute IS (59–62) and with mortality in acute HS (62, 63), and the normalization of blood glucose appeared to cause a potential survival benefit (64). However, another report suggests that the relationship between hyperglycemia and poor outcome may reflect stress response in acute stroke instead of a deleterious effect of glucose (65). Our study revealed that high acute-phase glucose was associated with high 30-day and 1-year mortality in both IS and HS.

Our study also found that low diastolic BP and mean BP at admission were significantly associated with high 30-day and 1-year mortality, and low systolic BP was associated with 1-year mortality in acute IS. Previous studies showed the U-shape effect of BP on mortality in patients with IS (66–68). In addition, low diastolic BP seemed to escalate the risk for mortality more consistently than low systolic BP (62), which was similar to our finding.

## Limitations and strengths

We recognized that our study had limitations. First, our study was limited to elderly Han-Chinese patients, and there is a concern regarding the applicability of these findings in young patients and other ethnic populations. Second, some specific lipoproteins such as small dense LDL, electronegative LDL, and lipoprotein (a) were not measured, and we could not verify the operating mechanisms to explain the identified risk associations. Also, since we studied only statin-naïve stroke patients, it is likely that a large proportion of subjects in the highest part of lipid distribution have been excluded. So, the conclusions about stroke risk are conditional to patients having low to normal lipid levels. Third, we did not examine the association between functional outcome such as MRS and lipid levels, because SRICHS only evaluated short-term MRS at 3 months. Previous epidemiological reports in Japan (5–10) have demonstrated that there was significant difference in the outcome if the study was done with a long period of follow-up. Instead, we linked our SRICHS to national TDR to study the stroke outcome for a long period and to secure good reliability with no missing results. Fourth, in our study of HS, lobar hemorrhage was excluded because of the fear of a high prevalence of vascular anomalies. Additional reasons to exclude lobar hemorrhage were [1] a previous study that showed elevated HDL and decreased TG levels were significantly associated with an increased risk for lobar cerebral microbleeds (CMBs) but not for deep CMBs (69); [2] a study revealed that APOE epsilon4 was associated with recurrent HS that was unrelated to cerebral amyloid angiopathy, and it revealed that circulating lipid levels might mediate the role of APOE epsilon4 in the nonlobar HS (70); [3] a recent study examining a different cohort in Taiwan, which included both lobar and nonlobar HS, demonstrated that low TC level was associated with greater neurological severity and worse 3-month outcomes (39). Since these previous studies showed the controversial effect of lipids on cerebral hemorrhage, the present study intended to exclude lobar hemorrhage and focused the lipid study on nonlobar cerebral hemorrhage. There was concern that deep hemorrhage could also be related to vascular anomaly. However, first, the hematoma shape and location could be somewhat different from deep hemorrhage not related to vascular anomaly, and cerebral angiography was usually performed in our hospitals for further confirmation. Second, according to our previous studies in young patients with cerebral hemorrhage, vascular anomaly of 16.9% was seen in all cerebral hemorrhages (71) and only 5.3% was seen in cerebral deep hemorrhage (72), which suggests that vascular anomaly could also be rare in elderly patients with deep cerebral hemorrhage. The above reasons may suggest that vascular anomaly could be rare in our study patients with deep hemorrhage.

The strength of our study is that the selection of patients was strict to prevent selection bias. First, to eliminate the influence of lipid-lowering agents, we excluded those patients who were known or uncertain about the use of lipid-lowering agents prior to index stroke. Second, since remarkable change of serum lipid levels can be seen in the early phase after acute IS onset (46), and since serum TC level may decrease soon after an acute stroke (52–55, 56) and then increase to the highest level at 3 months, we included only those patients who had a stroke with the onset of symptoms ≤1 day for examining the acute-phase lipid levels in stroke patients. Third, we recruited only those patients who had their blood samples measured for lipids the first working morning after admission to hospital. To confirm the reliability of acute-phase laboratory blood tests, we did the sensitivity analysis of laboratory findings between sampling within 12 h and 12–24 h (Supplemental Tables 5-1, 5-2).

## Conclusion

Lipid paradox can be seen in statin-naïve acute IS but not in hypertension-related HS with low acute-phase lipid levels being associated with high mortality. Owing to the small sample size of HS, further studies with larger sample size may be needed. The mortality in IS increased when low lipid levels were incorporated with high fasting glucose, low BP, and high NIHSS score.

## Availability of data and materials

The data that support the findings of this study are available from the Stroke Center, Linkou Chang Gung Memorial Hospital, Taoyuan, Taiwan, but restrictions apply to the availability of these data, which were used under the approval of Institutional Review Board of Linkou Chang Gung Memorial Hospital for current study and so are not publicly available. However, data will be made available by the authors upon reasonable request and with the permission of the Institutional Review Board of Linkou Chang-Gung Memorial Hospital.

## Author contributions

K-HC helped in generating original ideas in study design and analysis of data, in drafting of the manuscript, in revising it critically for important intellectual content, and in final approval of the manuscript submitted. J-RL helped with data acquisition and statistical analysis. CA helped in drafting the manuscript, revising it critically for important intellectual content, and in the final approval of the manuscript submitted. W-TL contributed to the conception, design, analysis, and interpretation of data; drafting of the manuscript; and revising it critically for important intellectual content. T-HL contributed to the conception, design, analysis, and interpretation of the data; drafting of the manuscript; revising it critically for important intellectual content; and the final approval of the manuscript submitted.

## Conflict of interest statement

The authors declare that the research was conducted in the absence of any commercial or financial relationships that could be construed as a potential conflict of interest.

## Abbreviations

BP: blood pressure
HDL: high-density lipoprotein
HS: hemorrhagic stroke
IS: ischemic stroke
LDL: low-density lipoprotein
NIHSS: National Institutes of Health Stroke Scale
Non-HDL-C: non-high-density lipoprotein cholesterol
SRICHS: Stroke Registry In Chang-Gung Healthcare System
TC: total cholesterol
UA: uric acid.
