# Supplementary material for: Lipid Paradox in Statin-Naïve Acute Ischemic Stroke But Not Hemorrhagic Stroke
Source: Front Neurol. 2018 Aug 29;9:541. doi: 10.3389/fneur.2018.00541 (PMC6124481; doi:10.3389/fneur.2018.00541)
Supplement: Supplementary file 1 [file Table_1.DOCX]

| **Supplemental table 1.**  **The effect of lipid levels on outcome between ischemic (IS) and hemorrhagic stroke (HS)** | | | | | | |
| --- | --- | --- | --- | --- | --- | --- |
| 30-day mortality, n (%) | | | | | | |
| Stroke subtype | Low TC | High TC | Odds ratio | 95% CI | | p value for IS and HS interaction |
| IS | 80 (5.96) | 49 (2.39) | 2.59 | 1.86 | 3.62 | 0.032 |
| HS | 30 (25.1) | 6 (6.19) | 5.07 | 3.03 | 8.49 |  |
|  | Low TG | High TG |  |  |  | 0.097 |
| IS | 83 (5.51) | 46 (2.42) | 2.35 | 1.68 | 3.30 |  |
| HS | 30 (23.2) | 6 (7.19) | 3.89 | 2.34 | 6.45 |  |
|  | Low LDL | High LDL |  |  |  |  |
| IS | 76 (5.22) | 53 (2.71) | 1.98 | 1.42 | 2.75 | 0.017 |
| HS | 31 (23.1) | 5 (6.69) | 4.20 | 2.48 | 7.11 |  |
|  | Low N-HDL-C | High N-HDL-C |  |  |  |  |
| IS | 90 (5.33) | 39 (2.28) | 2.42 | 1.70 | 3.44 | 0.315 |
| HS | 31 (21.3) | 5 (7.52) | 3.33 | 1.94 | 5.71 |  |
|  | Low TC/HDL | High TC/HDL |  |  |  |  |
| IS | 84 (5.10) | 45 (2.57) | 2.03 | 1.45 | 2.85 | 0.043 |
| HS | 32 (21.4) | 4 (6.22) | 3.57 | 2.02 | 6.29 |  |
|  |  |  |  |  |  |  |
| 1-year mortality | | | | | | |
| Stroke subtype | Low TC | High TC | Odds ratio | 95% CI | | p value for IS and HS interaction |
| IS | 219 (15.3) | 189 (8.56) | 1.93 | 1.58 | 2.35 | <0.001 |
| HS | 44 (33.7) | 9 (9.12) | 5.06 | 3.26 | 7.85 |  |
|  | Low TG | High TG |  |  |  | 0.001 |
| IS | 240 (14.8) | 168 (8.30) | 1.93 | 1.58 | 2.35 |  |
| HS | 44 (31.7) | 9 (9.71) | 4.30 | 2.76 | 6.72 |  |
|  | Low LDL | High LDL |  |  |  |  |
| IS | 224 (14.3) | 184 (8.84) | 1.73 | 1.42 | 2.10 | <0.001 |
| HS | 45 (31.2) | 8 (9.67) | 4.24 | 2.70 | 6.67 |  |
|  | Low N-HDL-C | High N-HDL-C |  |  |  |  |
| IS | 264 (14.6) | 144 (7.82) | 2.02 | 1.65 | 2.48 | 0.043 |
| HS | 45 (28.9) | 8 (10.6) | 3.42 | 2.15 | 5.45 |  |
|  | Low TC/HDL | High TC/HDL |  |  |  |  |
| IS | 246 (14.1) | 162 (8.51) | 1.77 | 1.45 | 2.16 | 0.021 |
| HS | 46 (28.4) | 7 (10.7) | 3.29 | 2.05 | 5.29 |  |
| TC= Total cholesterol, TG= Triglyceride, LDL= Low-density lipoprotein, HDL= High-density lipoprotein, N-HDL-C= Non-HDL cholesterol, TC/HDL= Total cholesterol/HDL ratio.  Statistical analysis: Logistic regression with no adjustment of any confounding factor. | | | | | | |

**Supplemental table 2. The correlation between risk factors and lipid levels for 30-day mortality in first-ever, statin-naïve acute ischemic stroke**

| Total cholesterol (TC) | | | | | |
| --- | --- | --- | --- | --- | --- |
| Risk factor, n (%) | Low TC | High TC | p value | TC level (mean±SD) | p value |
| Hypertension |  |  | 0.003 |  | <0.001 |
| No | 430 (28.6) | 564 (24.5) |  | 177.1±43.4 |  |
| Yes | 1,049 (71.4) | 1,703 (75.5) |  | 183.0±42.5 |  |
| Diabetes mellitus |  |  | 0.638 |  | 0.230 |
| No | 963 (64.5) | 1,532 (66.9) |  | 180.8±41.4 |  |
| Yes | 515 (35.5) | 736 (33.1) |  | 182.7±45.6 |  |
| Atrial fibrillation |  |  | <0.001 |  | <0.001 |
| No | 1,103 (74.9) | 1,913 (84.4) |  | 184.7±43.5 |  |
| Yes | 373 (25.1) | 357 (15.6) |  | 168.3±37.3 |  |
|  |  |  |  |  |  |

| Triglyceride (TG) | | | | | |
| --- | --- | --- | --- | --- | --- |
| Risk factor, n (%) | Low TG | High TG | p value | TG level (mean±SD) | p value |
| Hypertension |  |  | <0.001 |  | <0.001 |
| No | 515 (30.5) | 479 (22.7) |  | 118.4±85.6 |  |
| Yes | 1,149 (69.5) | 1,603 (77.3) |  | 133.8±100 |  |
| Diabetes mellitus |  |  | <0.001 |  | <0.001 |
| No | 1,261 (75.1) | 1,234 (58.7) |  | 118.0±91.9 |  |
| Yes | 406 (24.9) | 845 (41.3) |  | 152.9±102 |  |
| Atrial fibrillation |  |  | <0.001 |  | <0.001 |
| No | 1,198 (72.2) | 1,818 (87.4) |  | 137.2±102 |  |
| Yes | 465 (27.8) | 265 (12.6) |  | 99.6±61.6 |  |

|  |  |  |  |  |  |
| --- | --- | --- | --- | --- | --- |
| Low-density lipoprotein (LDL) | | | | | |
| Risk factor, n (%) | Low LDL | High LDL | p value | LDL level (mean±SD) | p value |
| Hypertension |  |  | 0.103 |  | 0.002 |
| No | 450 (27.4) | 544 (25.2) |  | 110.7±35.6 |  |
| Yes | 1,166 (72.6) | 1,586 (74.8) |  | 114.8±35.9 |  |
| Diabetes mellitus |  |  | 0.006 |  | 0.616 |
| No | 1,037 (63.6) | 1,458 (67.7) |  | 113.9±34.3 |  |
| Yes | 577 (36.4) | 674 (32.3) |  | 113.3±38.7 |  |
| Atrial fibrillation |  |  | <0.001 |  | <0.001 |
| No | 1,234 (76.7) | 1,782 (83.6) |  | 116.0±36.3 |  |
| Yes | 378 (23.3) | 352 (16.4) |  | 104.5±32.4 |  |
|  |  |  |  |  |  |
| Non-high-density lipoprotein cholesterol (N-HDL-C) | | | | | |
| Risk factor, n (%) | Low N-HDL-C | High N-HDL-C | p value | N-HDL-C level (mean±SD) | p value |
| Hypertension |  |  | 0.001 |  | <0.001 |
| No | 537 (28.3) | 457 (23.9) |  | 133.4±42.1 |  |
| Yes | 1,326 (71.7) | 1,426 (76.1) |  | 139.8±41.3 |  |
| Diabetes mellitus |  |  | 0.151 |  | <0.001 |
| No | 1,261 (67.0) | 1,234 (64.9) |  | 136.2±40.3 |  |
| Yes | 602 (33.0) | 649 (35.1) |  | 141.8±43.7 |  |
| Atrial fibrillation |  |  | <0.001 |  | <0.001 |
| No | 1,380 (74.4) | 1,636 (86.8) |  | 141.7±42.0 |  |
| Yes | 479 (25.6) | 251 (13.2) |  | 123.2±36.3 |  |
|  |  |  |  |  |  |
| Total cholesterol/High-density lipoprotein ratio (TC/HDL) | | | | | |
| Risk factor, n (%) | Low TC/HDL | High TC/HDL | p value | TC/HDL level (mean±SD) | p value |
| Hypertension |  |  | <0.001 |  | 0.002 |
| No | 529 (28.6) | 465 (23.8) |  | 4.32±1.53 |  |
| Yes | 1,292 (71.4) | 1,460 (76.2) |  | 4.51±1.75 |  |
| Diabetes mellitus |  |  | <0.001 |  | <0.001 |
| No | 1,299 (70.7) | 1,196 (61.5) |  | 4.33±1.80 |  |
| Yes | 524 (29.3) | 727 (38.5) |  | 4.72±1.44 |  |
| Atrial fibrillation |  |  | <0.001 |  | <0.001 |
| No | 1,363 (75.3) | 1,653 (85.7) |  | 4.58±1.75 |  |
| Yes | 452 (24.7) | 278 (14.3) |  | 4.00±1.35 |  |
| Cut-off points of TC, TG, LDL, N-HDL-C and TC/HDL ratio= 163.5 mg/dL, 94.5 mg/dL, 100 mg/dL, 130.5 mg/dL and 4.06 for 30-day mortality.  Statistical analysis: Mean±SD for continuous variables using t-test and numbers (%) for categorical variables using chi-square test. | | | | | |

**Supplemental table 3-1. Clinical findings and hazard ratio of 30-day and 1-year mortality in first-ever, statin-naïve acute ischemic stroke using continuous data**

|  | Ischemic stroke- 30-day | | |  | | | | Ischemic stroke- 1-year | | |  | | | |
| --- | --- | --- | --- | --- | --- | --- | --- | --- | --- | --- | --- | --- | --- | --- |
| Clinical findings and NIHSS | Survival | Mortality | P value | HR | 95% CI | | P value | Survival | Mortality | P value | HR | 95% CI | | P value |
|  | n=3,617 | n=129 |  |  | Lower | Upper |  | n=3,338 | n=408 |  |  | Lower | Upper |  |
| Total cholesterol (mg/dL) | 179 (154,206) | 160 (133,198) | <0.001* | 1.00 | 1.00 | 1.00 | 0.768 | 180 (155,207) | 166 (136,195) | <0.001* | 1.00 | 0.99 | 1.00 | 0.010 |
| Triglyceride (mg/dL) | 108 (78,155) | 88 (59,117) | <0.001* | 1.00 | 1.00 | 1.00 | 0.369 | 110 (79,157) | 89 (66,119) | <0.001* | 1.00 | 1.00 | 1.00 | 0.295 |
| Low-density lipoprotein (LDL; mg/dL) | 111 (90,134) | 100 (74,129) | 0.004* | 1.00 | 0.99 | 1.00 | 0.712 | 112 (91,135) | 101 (79,126) | <0.001* | 1.00 | 0.99 | 1.00 | 0.021 |
| High-density lipoprotein (HDL; mg/dL) | 41 (35,50) | 43 (33,53) | 0.835 | 1.00 | 0.98 | 1.01 | 0.662 | 41 (35,50) | 42 (33,52) | 0.914 | 1.00 | 0.99 | 1.01 | 0.577 |
| Non-HDL cholesterol (mg/dL) | 135 (111,162) | 116 (91,150) | <0.001* | 1.00 | 1.00 | 1.00 | 0.812 | 136 (113,162) | 119 (94,149) | <0.001* | 1.00 | 0.99 | 1.00 | 0.015 |
| Total cholesterol/HDL ratio | 4.3 (3.4,5.2) | 3.8 (3.2,5.0) | 0.009* | 1.12 | 1.07 | 1.16 | <0.001 | 4.3 (3.5,5.2) | 3.8 (3.1,4.9) | <0.001* | 1.06 | 1.00 | 1.13 | 0.062 |
| Systolic blood pressure (mmHg) | 151 (134,170) | 147 (128,174) | 0.164 | 1.00 | 0.99 | 1.00 | 0.097 | 151 (135,170) | 148 (130,170) | 0.009* | 1.00 | 0.99 | 1.00 | 0.033 |
| Diastolic blood pressure (mmHg) | 85 (76,96) | 79 (67,95) | <0.001* | 0.98 | 0.97 | 0.99 | <0.001 | 86 (77,96) | 80 (70,93) | <0.001* | 0.99 | 0.98 | 0.99 | <0.001 |
| Mean blood pressure (mmHg) | 108 (97,120) | 103 (88,119) | 0.008* | 0.99 | 0.98 | 1.00 | 0.004 | 108 (97,121) | 103 (91,117) | <0.001* | 0.99 | 0.99 | 1.00 | <0.001 |
| Fasting glucose (mg/dL) | 114 (95,152) | 148 (113,190) | <0.001* | 1.02 | 1.00 | 1.01 | <0.001 | 114 (94,151) | 133 (106,181) | <0.001* | 1.00 | 1.00 | 1.01 | <0.001 |
| Uric acid (mg/dL) | 5.5 (4.5,6.6) | 5.4 (4.0,7.0) | 0.608 | 0.97 | 0.87 | 1.08 | 0.520 | 5.5 (4.5,6.6) | 5.7 (4.1,7.0) | 0.890 | 0.98 | 0.92 | 1.04 | 0.425 |
| NIHSS score | 4.0 (2.0,9.0) | 20 (14,29) | <0.001* | 1.11 | 1.1.0 | 1.13 | <0.001 | 4.0 (2.0,8.0) | 14 (6.0,22) | <0.001* | 1.08 | 1.07 | 1.09 | <0.001 |

*p<0.05. NIHSS= national institutes of health stroke scale. HR= hazard ratio, CI= confidence interval. Median (Q1,Q3) are reported for continuous variables using Mann-Whitney U test.

Multivariate Cox proportional hazard model after adjusting age, sex, age × sex (interaction), atrial fibrillation, smoking, alcohol, hypertension, diabetes mellitus, and family history of coronary artery disease for 30-day mortality; age, sex, age × sex (interaction), atrial fibrillation, smoking, alcohol, hypertension, diabetes mellitus, family history of coronary artery disease and family history of stroke for 1-year mortality.

**Supplemental table 3-2. Clinical findings and hazard ratio of 30-day and 1-year mortality in first-ever, statin-naïve acute intracerebral hemorrhagic stroke using continuous data**

|  | Hemorrhagic stroke- 30-day | | |  | | | | Hemorrhagic stroke- 1-year | | |  | | | |
| --- | --- | --- | --- | --- | --- | --- | --- | --- | --- | --- | --- | --- | --- | --- |
| Clinical findings and NIHSS | Survival | Mortality | P value | HR | 95% CI | | P value | Survival | Mortality | P value | HR | 95% CI | | P value |
|  | n=429 | n=36 |  |  | Lower | Upper |  | n=412 | n=53 |  |  | Lower | Upper |  |
| Total cholesterol (mg/dL) | 180 (153,206) | 168 (139,208) | 0C76 | 1.00 | 1.00 | 1.01 | 0.381 | 180 (154,206) | 167 (143,208) | 0.196 | 1.00 | 0.99 | 1.01 | 0.681 |
| Triglyceride (mg/dL) | 106 (79,148) | 103 (79,174) | 0.942 | 1.00 | 1.00 | 1.00 | 0.839 | 107 (80,148) | 97 (75,143) | 0.363 | 1.00 | 1.00 | 1.00 | 0.634 |
| Low-density lipoprotein (LDL; mg/dL) | 110 (86,132) | 101 (74,141) | 0.573 | 1.00 | 0.99 | 1.01 | 0.633 | 110 (86,132) | 101 (75,138) | 0.572 | 1.00 | 0.99 | 1.01 | 0.860 |
| High-density lipoprotein (HDL; mg/dL) | 45 (37,55) | 48 (40,52) | 0.816 | 1.00 | 0.97 | 1.02 | 0.676 | 45 (37,55) | 47 (37,53) | 0.766 | 0.99 | 0.97 | 1.01 | 0.446 |
| Non-HDL cholesterol (mg/dL) | 130 (106,160) | 124 (96,162) | 0.453 | 1.00 | 0.99 | 1.01 | 0.643 | 130 (106,160) | 126 (96,162) | 0.395 | 1.00 | 0.99 | 1.01 | 0.923 |
| Total cholesterol/HDL ratio | 3.9 (3.1,4.9) | 3.8 (3.1,4.9) | 0.968 | 1.03 | 0.92 | 1.15 | 0.586 | 3.9 (3.1,4.9) | 3.9 (3.1,4.9) | 0.908 | 1.04 | 0.95 | 1.14 | 0.400 |
| Systolic blood pressure (mmHg) | 157 (142,171) | 161 (129,195) | 0.582 | 0.99 | 0.98 | 1.00 | 0.001 | 156 (141,171) | 158 (137,188) | 0.521 | 0.99 | 0.98 | 1.00 | <0.001 |
| Diastolic blood pressure (mmHg) | 89 (80,100) | 86 (71,106) | 0.584 | 0.98 | 0.97 | 0.99 | <0.001 | 90 (80,100) | 85 (72,105) | 0.260 | 0.98 | 0.97 | 0.99 | <0.001 |
| Mean blood pressure (mmHg) | 112 (101,124) | 115 (93,134) | 0.918 | 0.98 | 0.97 | 0.99 | <0.001 | 112 (102,124) | 109 (95,132) | 0.817 | 0.98 | 0.98 | 0.99 | <0.001 |
| Fasting glucose (mg/dL) | 109 (96,137) | 163 (141,238) | <0.001* | 1.01 | 1.01 | 1.01 | <0.001 | 108 (96,136) | 149 (130,236) | <0.001* | 1.01 | 1.01 | 1.01 | <0.001 |
| Uric acid (mg/dL) | 5.5 (4.4,6.8) | 6.7 (5.7,9.6) | 0.005* | 1.60 | 1.33 | 1.92 | <0.001 | 5.5 (4.4,6.8) | 6.1 (5.0,7.9) | 0.027* | 1.41 | 1.20 | 1.65 | <0.001 |
| NIHSS score | 8.0 (4.0,14) | 38 (38,40) | <0.001* | 1.14 | 1.12 | 1.17 | <0.001 | 7.5 (3.0,14) | 38 (20,40) | <0.001* | 1.11 | 1.10 | 1.13 | <0.001 |

*p<0.05. NIHSS= national institutes of health stroke scale. HR= hazard ratio, CI= confidence interval. Median (Q1,Q3) are reported for continuous variables using Mann-Whitney U test.

Multivariate Cox proportional hazard model after adjusting age, sex, hypertension, and diabetes mellitus for 30-day and 1-year mortality in hemorrhagic stroke.

| **Supplementary table 4-a. Pearson correlations between parameters in ischemic stroke at 30 days** | | | | | | | | |
| --- | --- | --- | --- | --- | --- | --- | --- | --- |
|  | **TC** | **TG** | **LDL** | **HDL** | **Non-HDL-C** | **TC/HDL** | **Glucose** | **UA** |
| **TC (p value)** | r=1 | r=0.71032 | r=0.86176 | r=0.25019 | r=0.97425 | r=0.63694 | r=0.01529 | r=-0.11989 |
|  |  | <0.0001* | <0.0001* | 0.0084* | <0.0001* | <0.0001* | 0.8837 | 0.2773 |
| **TG (p value)** | r=0.71032 | r=1 | r=0.33277 | r=-0.28204 | r=0.80391 | r=0.94394 | r=-0.03391 | r=-0.04646 |
|  | <0.0001* |  | 0.0004* | 0.0028* | <0.0001* | <0.0001* | 0.7456 | 0.6747 |
| **LDL (p value)** | r=0.86176 | r=0.33277 | r=1 | r=0.29993 | r=0.82055 | r=0.29434 | r=0.05715 | r=-0.12235 |
|  | <0.0001* | 0.0004* |  | 0.0015* | <0.0001* | 0.0019* | 0.5906 | 0.2765 |
| **HDL (p value)** | r=0.25019 | r=-0.28204 | r=0.29993 | r=1 | r=0.02544 | r=-0.41907 | r=-0.06258 | r=-0.07482 |
|  | 0.0084* | 0.0028* | 0.0015* |  | 0.792 | <0.0001* | 0.5534 | 0.5041 |
| **Non-HDL-C (p value)** | r=0.97425 | r=0.80391 | r=0.82055 | r=0.02544 | r=1 | r=0.75524 | r=0.00647 | r=-0.09586 |
|  | <0.0001* | <0.0001* | <0.0001* | 0.792 |  | <0.0001* | 0.9512 | 0.3916 |
| **TC/HDL**  **(p value)** | r=0.63694 | r=0.94394 | r=0.29434 | r=-0.41907 | r=0.75524 | r=1 | r=-0.07478 | r=-0.05448 |
|  | <0.0001* | <0.0001* | 0.0019* | <0.0001* | <0.0001* |  | 0.4787 | 0.6269 |
| **Glucose**  **(p value)** | r=0.01529 | r=-0.03391 | r=0.05715 | r=-0.06258 | r=0.00647 | r=-0.07478 | r=1 | r=0.02347 |
|  | 0.8837 | 0.7456 | 0.5906 | 0.5534 | 0.9512 | 0.4787 |  | 0.8426 |
| **UA (p value)** | r=-0.11989 | r=-0.04646 | r=-0.12235 | r=-0.07482 | r=-0.09586 | r=-0.05448 | r=0.02347 | r=1 |
|  | 0.2773 | 0.6747 | 0.2765 | 0.5041 | 0.3916 | 0.6269 | 0.8426 |  |
| *p<0.05, TC= total cholesterol, FG= fasting glucose, LDL= Low-density lipoprotein, HDL= High-density lipoprotein, Non-HDL-C= Non-HDL cholesterol, TC/HDL= total cholesterol/HDL ratio, UA= uric acid. | | | | | | | | |

| **Supplementary table 4-b. Pearson correlations between parameters in ischemic stroke at 1 year** | | | | | | | | |
| --- | --- | --- | --- | --- | --- | --- | --- | --- |
|  | **TC** | **TG** | **LDL** | **HDL** | **Non-HDL-C** | **TC/HDL** | **Glucose** | **UA** |
| **TC (p value)** | r=1 | r=0.29828 | r=0.93995 | r=0.24148 | r=0.94438 | r=0.47051 | r=-0.0585 | r=0.08194 |
|  |  | <0.0001* | <0.0001* | 0.0001* | <0.0001* | <0.0001* | 0.4328 | 0.2585 |
| **TG (p value)** | r=0.29828 | r=1 | r=0.17174 | r=-0.41747 | r=0.44518 | r=0.59652 | r=0.32738 | r=0.20917 |
|  | <0.0001* |  | 0.0068* | <0.0001* | <0.0001* | <0.0001* | <0.0001* | 0.0036* |
| **LDL (p value)** | r=0.93995 | r=0.17174 | r=1 | r=0.03771* | r=0.95181 | r=0.58284 | r=-0.10899 | r=0.07192 |
|  | <0.0001 | 0.0068* |  | 0.5553 | <0.0001 | <0.0001* | 0.1453 | 0.3267 |
| **HDL (p value)** | r=0.24148 | r=-0.41747 | r=0.03771 | r=1 | r=-0.09108 | r=-0.67455 | r=-0.11688 | r=-0.13668 |
|  | 0.0001* | <0.0001* | 0.5553 |  | 0.1535 | <0.0001* | 0.1182 | 0.0614 |
| **Non-HDL-C (p value)** | r=0.94438 | r=0.44518 | r=0.95181 | r=-0.09108 | r=1 | r=0.71145 | r=-0.01214* | r=0.14796 |
|  | <0.0001* | <0.0001* | <0.0001* | 0.1535 |  | <0.0001* | 0.8715 | 0.0427* |
| **TC/HDL**  **(p value)** | r=0.47051 | r=0.59652 | r=0.58284 | r=-0.67455 | r=0.71145 | r=1 | r=0.08031 | r=0.16752 |
|  | <0.0001* | <0.0001* | <0.0001* | <0.0001* | <0.0001* |  | 0.2838 | 0.0216 |
| **Glucose**  **(p value)** | r=-0.0585 | r=0.32738 | r=-0.10899 | r=-0.11688 | r=-0.01214 | r=0.08031 | r=1 | r=0.13738 |
|  | 0.4328 | <0.0001* | 0.1453 | 0.1182 | 0.8715 | 0.2838 |  | 0.1107 |
| **UA (p value)** | r=0.08194 | r=0.20917 | r=0.07192 | r=-0.13668 | r=0.14796 | r=0.16752 | r=0.13738 | r=1 |
|  | 0.2585 | 0.0036* | 0.3267 | 0.0614 | 0.0427* | 0.0216* | 0.1107 |  |
| *p<0.05, TC= total cholesterol, FG= fasting glucose, LDL= Low-density lipoprotein, HDL= High-density lipoprotein, Non-HDL-C= Non-HDL cholesterol, TC/HDL= total cholesterol/HDL ratio, UA= uric acid. | | | | | | | | |

| **Supplementary table 4-c. Pearson correlations between parameters in hemorrhagic stroke at 30 days** | | | | | | | | |
| --- | --- | --- | --- | --- | --- | --- | --- | --- |
|  | **TC** | **TG** | **LDL** | **HDL** | **Non-HDL-C** | **TC/HDL** | **Glucose** | **UA** |
| **TC (p value)** | r=1 | r=0.20935 | r=0.92414 | r=0.55821 | r=0.95371 | r=-0.19826 | r=-0.04696 | r=-0.19538 |
|  |  | 0.1579 | <0.0001* | <0.0001* | <0.0001* | 0.1816 | 0.8315 | 0.3098 |
| **TG (p value)** | r=0.20935 | r=1 | r=-0.04447 | r=-0.05012 | r=0.26018 | r=0.23548 | r=0.05373 | r=0.1961 |
|  | 0.1579 |  | 0.7666 | 0.738 | 0.0774 | 0.1111 | 0.8076 | 0.3079 |
| **LDL (p value)** | r=0.92414 | r=-0.04447 | r=1 | r=0.32987 | r=0.94514 | r=-0.0561 | r=-0.01589 | r=-0.18602 |
|  | <0.0001* | 0.7666 |  | 0.022* | <0.0001* | 0.708 | 0.9413 | 0.325 |
| **HDL (p value)** | r=0.55821 | r=-0.05012 | r=0.32987 | r=1 | r=0.28286 | r=-0.74303 | r=0.01035 | r=-0.42125 |
|  | <0.0001* | 0.738 | 0.022 |  | 0.054 | <0.0001* | 0.9617 | 0.0204* |
| **Non-HDL-C (p value)** | r=0.95371 | r=0.26018 | r=0.94514 | r=0.28286 | r=1 | r=0.04011 | r=-0.05309 | r=-0.0844 |
|  | <0.0001* | 0.0774 | <0.0001 | 0.054 |  | 0.7889 | 0.8099 | 0.6634 |
| **TC/HDL**  **(p value)** | r=-0.19826 | r=0.23548 | r=-0.0561 | r=-0.74303 | r=0.04011 | r=1 | r=-0.13271 | r=0.34562 |
|  | 0.1816 | 0.1111 | 0.708 | <0.0001 | 0.7889 |  | 0.5461 | 0.0663 |
| **Glucose**  **(p value)** | r=-0.04696 | r=0.05373 | r=-0.01589 | r=0.01035 | r=-0.05309 | r=-0.13271 | r=1 | r=-0.38505 |
|  | 0.8315 | 0.8076 | 0.9413 | 0.9617 | 0.8099 | 0.5461 |  | 0.127 |
| **UA (p value)** | r=-0.19538 | r=0.1961 | r=-0.18602 | r=-0.42125 | r=-0.0844 | r=0.34562 | r=-0.38505 | 1 |
|  | 0.3098 | 0.3079 | 0.325 | 0.0204 | 0.6634 | 0.0663 | 0.127 |  |
| *p<0.05, TC= total cholesterol, FG= fasting glucose, LDL= Low-density lipoprotein, HDL= High-density lipoprotein, Non-HDL-C= Non-HDL cholesterol, TC/HDL= total cholesterol/HDL ratio, UA= uric acid. | | | | | | | | |

| **Supplementary table 4-d. Pearson correlations between parameters in hemorrhagic stroke at 1 year** | | | | | | | | |
| --- | --- | --- | --- | --- | --- | --- | --- | --- |
|  | **TC** | **TG** | **LDL** | **HDL** | **Non-HDL-C** | **TC/HDL** | **Glucose** | **UA** |
| **TC (p value)** | r=1 | r=0.23571 | r=0.95808 | r=0.28787 | r=0.96113 | r=0.59758 | r=0.20725 | r=0.09393 |
|  |  | 0.1867 | <0.0001* | 0.1043 | <0.0001* | 0.0002* | 0.4586 | 0.6699 |
| **TG (p value)** | r=0.23571 | r=1 | r=0.18127 | r=-0.20817 | r=0.30612 | r=0.42989 | r=0.79454 | r=0.19761 |
|  | 0.1867 |  | 0.3208 | 0.245 | 0.0832 | 0.0125* | 0.0004* | 0.3661 |
| **LDL (p value)** | r=0.95808 | r=0.18127 | r=1 | r=0.07789 | r=0.98565 | r=0.72565 | r=0.09012 | r=0.02897 |
|  | <0.0001* | 0.3208 |  | 0.6718 | <0.0001* | <0.0001* | 0.7593 | 0.8956 |
| **HDL (p value)** | r=0.28787 | r=-0.20817 | r=0.07789 | r=1 | r=0.01228 | r=-0.56475 | r=0.0992 | r=0.08921 |
|  | 0.1043 | 0.245 | 0.6718 |  | 0.9459 | 0.0006* | 0.725 | 0.6856 |
| **Non-HDL-C (p value)** | r=0.96113 | r=0.30612 | r=0.98565 | r=0.01228 | r=1 | r=0.78677 | r=0.20047 | r=0.07423 |
|  | <0.0001* | 0.0832 | <0.0001* | 0.9459 |  | <0.0001* | 0.4738 | 0.7364 |
| **TC/HDL**  **(p value)** | r=0.59758 | r=0.42989 | r=0.72565 | r=-0.56475 | r=0.78677 | r=1 | r=0.25177 | r=-0.03202 |
|  | 0.0002* | 0.0125* | <0.0001* | 0.0006* | <0.0001 |  | 0.3653 | 0.8847 |
| **Glucose**  **(p value)** | r=0.20725 | r=0.79454 | r=0.09012 | r=0.0992 | r=0.20047 | r=0.25177 | r=1 | r=-0.02972 |
|  | 0.4586 | 0.0004* | 0.7593 | 0.725 | 0.4738 | 0.3653 |  | 0.9269 |
| **UA (p value)** | r=0.09393 | r=0.19761 | r=0.02897 | r=0.08921 | r=0.07423 | r=-0.03202 | r=-0.02972 | r=1 |
|  | 0.6699 | 0.3661 | 0.8956 | 0.6856 | 0.7364 | 0.8847 | 0.9269 |  |
| *p<0.05, TC= total cholesterol, FG= fasting glucose, LDL= Low-density lipoprotein, HDL= High-density lipoprotein, Non-HDL-C= Non-HDL cholesterol, TC/HDL= total cholesterol/HDL ratio, UA= uric acid. | | | | | | | | |

| **Supplemental table 5-1. Sensitivity analysis of laboratory findings in survival and mortality groups between sampling within 12 hours and 12-24 hours in ischemic stroke** | | | | | | | | | | | | | | |
| --- | --- | --- | --- | --- | --- | --- | --- | --- | --- | --- | --- | --- | --- | --- |
|  | Ischemic stroke- 30-day | | | | | | |  | Ischemic stroke- 1-year | | | | | |
|  | Survival | | |  | Mortality | |  |  | Survival | |  | Mortality | |  |
| Laboratory findings at admission | <12 hrs | | 12-24 hrs |  | <12 hrs | 12-24 hrs |  |  | <12 hrs | 12-24 hrs |  | <12 hrs | 12-24 hrs |  |
|  | n=2,847 | | n=770 | p value | n=111 | n=18 | p value |  | n=2,633 | n=705 | p value | n=325 | n=83 | p value |
| Low TC (<163.5/163.5 mg/dL) | | 970 (34.2) | 257 (33.5) | 0.720 | 58 (52.3) | 13 (72.2) | 0.114 |  | 867 (33.0) | 231 (32.9) | 0.937 | 161 (49.5) | 39 (47.0) | 0.678 |
| Low TG (<94.5/94.5 mg/dL) | | 1,131 (39.7) | 298 (38.7) | 0.606 | 61 (55.0) | 13 (72.2) | 0.169 |  | 1,018 (38.7) | 264 (37.5) | 0.555 | 174 (53.5) | 47 (56.6) | 0.614 |
| Low LDL (<100/100 mg/dL) | | 1,030 (36.7) | 264 (34.9) | 0.356 | 50 (45.9) | 12 (70.6) | 0.058 |  | 923 (35.6) | 239 (34.4) | 0.580 | 157 (48.9) | 37 (46.3) | 0.670 |
| Low non-HDL-C (<130.5/130.5 mg/dL) | | 1,285 (45.4) | 324 (42.5) | 0.153 | 65 (59.6) | 16 (88.9) | 0.017* |  | 1,155 (44.1) | 291 (41.6) | 0.236 | 195 (60.8) | 49 (60.5) | 0.967 |
| Low HDL (<39.8/43.2 mg/dL) | | 1,242 (43.8) | 319 (41.8) | 0.313 | 47 (43.1) | 6 (33.3) | 0.435 |  | 1,501 (57.2) | 396 (56.5) | 0.735 | 174 (54.2) | 41 (50.6) | 0.563 |
| Low TC/HDL ratio (<4.06/4.04) | | 1,237 (43.7) | 332 (43.6) | 0.939 | 61 (56.0) | 13 (72.2) | 0.195 |  | 1,103 (42.2) | 292 (41.8) | 0.859 | 175 (54.5) | 49 (60.5) | 0.333 |
| High fasting glucose (≥108.9/108.9 mg/dL) | | 1,115 (56.3) | 316 (57.1) | 0.728 | 73 (76.8) | 14 (82.4) | 0.852 |  | 1,011 (55.4) | 282 (56.4) | 0.689 | 177 (70.8) | 48 (68.6) | 0.718 |
| High uric acid (≥8/8 mg/dL) | | 222 (9.6) | 43 (6.9) | 0.034* | 11 (13.8) | 0 (0.0) | 0.305 |  | 198 (9.2) | 36 (6.3) | 0.025* | 35 (14.3) | 7 (10.6) | 0.431 |
| *p<0.05. TC= total cholesterol, TG= Triglyceride, LDL= Low-density lipoprotein, HDL= High-density lipoprotein, Non-HDL-C= Non-HDL cholesterol, TC/HDL= total cholesterol/HDL ratio, hrs= hours. Numbers (percentage) are reported for categorical variables using chi-square test. | | | | | | | | | | | | | | |

| **Supplemental table 5-2. Sensitivity analysis of laboratory findings in survival and mortality groups between sampling within 12 hours and 12-24 hours in intracerebral hemorrhagic stroke** | | | | | | | | | | | | | | |
| --- | --- | --- | --- | --- | --- | --- | --- | --- | --- | --- | --- | --- | --- | --- |
|  | Hemorrhagic stroke- 30-day | | | | | | |  | Hemorrhagic stroke- 1-year | | | | | |
|  | Survival | | |  | Mortality | |  |  | Survival | |  | Mortality | |  |
| Laboratory findings at admission | | <12 hrs | 12- 24 hrs |  | <12 hrs | 12- 24 hrs |  |  | <12 hrs | 12- 24 hrs |  | <12 hrs | 12- 24 hrs |  |
|  |  | n=298 | n=131 | p value | n=25 | n=11 | p value |  | n=289 | n=123 | p value | n=34 | n=19 | p value |
| Low TC (<200/200 mg/dL) | 196 (65.8) | | 91 (69.5) | 0.454 | 19 (76.0) | 5 (45.5) | 0.159 |  | 191 (66.1) | 83 (67.5) | 0.785 | 24 (70.6) | 13 (68.4) | 0.869 |
| Low TG (<150/150 mg/dL) | 226 (75.8) | | 99 (75.6) | 0.953 | 19 (76.0) | 7 (63.6) | 0.720 |  | 219 (75.8) | 92 (74.8) | 0.832 | 26 (76.5) | 14 (73.7) | 1.000 |
| Low LDL (<100/100 mg/dL) | 109 (37.5) | | 56 (43.4) | 0.249 | 12 (52.2) | 4 (36.4) | 0.388 |  | 107 (37.9) | 51 (41.8) | 0.465 | 14 (43.8) | 9 (50.0) | 0.670 |
| Low non-HDL-C (<130/130 mg/dL) | 141 (47.8) | | 72 (55.0) | 0.172 | 14 (58.3) | 4 (36.4) | 0.227 |  | 137 (47.9) | 66 (53.7) | 0.286 | 18 (54.6) | 10 (52.6) | 0.894 |
| Low HDL (<45/45 mg/dL) | 148 (50.2) | | 59 (45.0) | 0.328 | 10 (41.7) | - ^†^ | 0.053 |  | 142 (49.7) | 56 (45.5) | 0.444 | 16 (48.5) | 4 (21.1) | 0.050 |
| Low TC/HDL ratio (<5/5) | 224 (75.9) | | 104 (79.4) | 0.434 | 18 (75.0) | 8 (72.7) | 0.746 |  | 218 (76.2) | 97 (78.9) | 0.561 | 24 (72.7) | 15 (79.0) | 0.868 |
| High fasting glucose (≥128.5/128.5 mg/dL) | 41 (27.7) | | 20 (34.5) | 0.338 | 9 (81.8) | 6 (100) | 1.000 |  | 39 (27.3) | 18 (32.7) | 0.448 | 11 (68.8) | 8 (88.9) | 0.520 |
| High uric acid (≥8/8 mg/dL) | 26 (11.0) | | 9 (9.5) | 0.680 | 5 (3.3) | - ^†^ | 0.787 |  | 26 (11.3) | 9 (9.9) | 0.723 | 5 (25.0) | - ^†^ | 0.872 |
| *p<0.05. TC= total cholesterol, TG= Triglyceride, LDL= Low-density lipoprotein, HDL= High-density lipoprotein, Non-HDL-C= Non-HDL cholesterol, TC/HDL= total cholesterol/HDL ratio, hrs= hours. Numbers (percentage) are reported for categorical variables using chi-square test.  ^†^ indicates the number of patient= 1 or 2 | | | | | | | | | | | | | | |
