# Supplementary material for: Lipid Paradox in Statin-Naïve Acute Ischemic Stroke But Not Hemorrhagic Stroke
Source: Front Neurol. 2018 Aug 29;9:541. doi: 10.3389/fneur.2018.00541 (PMC6124481; doi:10.3389/fneur.2018.00541)

Supplemental figures

Ischemic stroke

Receiver operating characteristic (ROC) curves to identify the cut-off points of admission NIHSS score for predicting 30-day and 1-year mortality in first-ever ischemic stroke

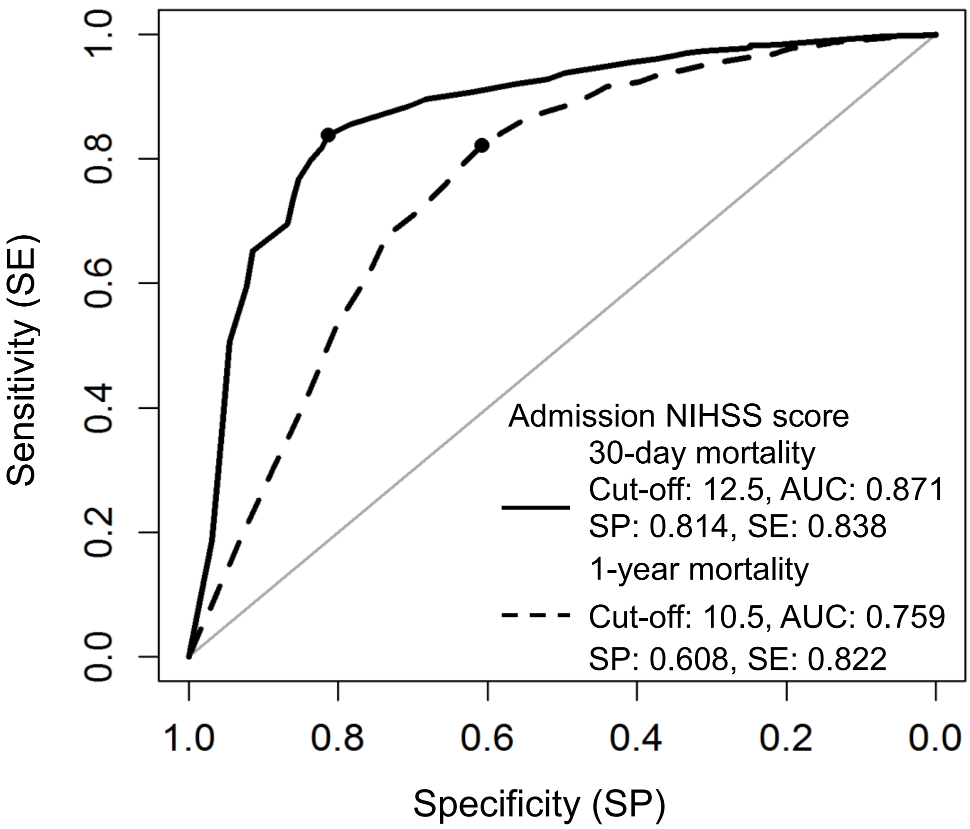

Receiver operating characteristic (ROC) curves to identify the cut-off points of admission total cholesterol (TC) level for predicting 30-day and 1-year mortality in first-ever ischemic stroke

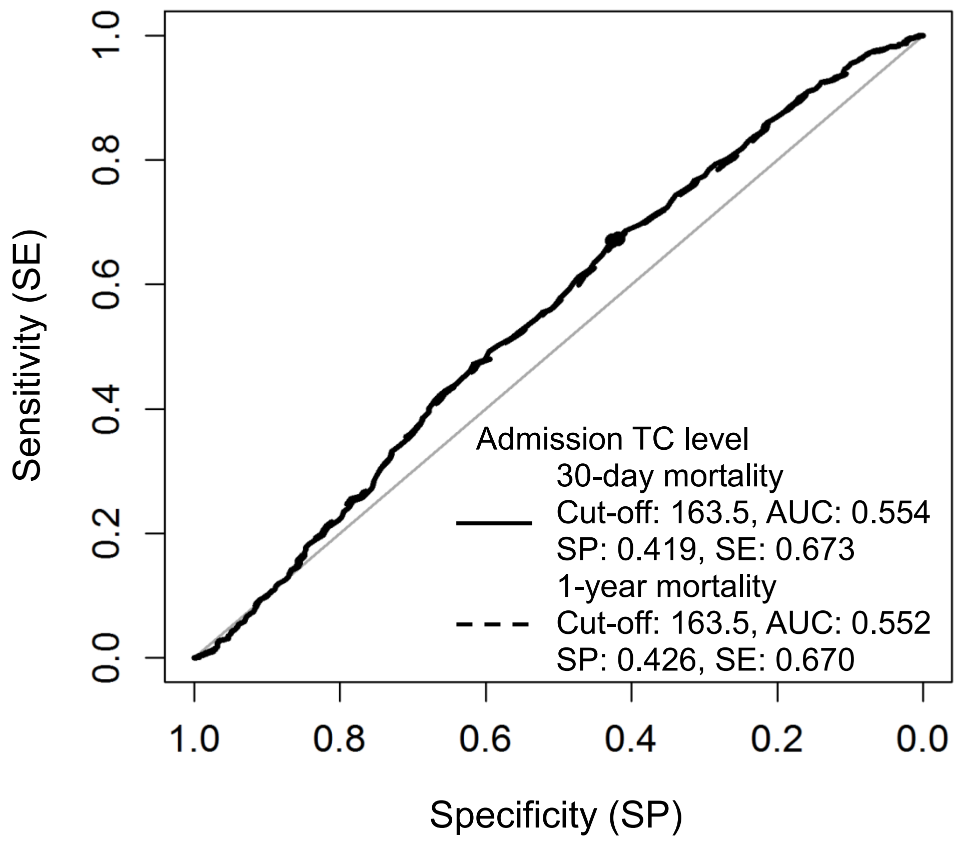

Receiver operating characteristic (ROC) curves to identify the cut-off points of admission triglyceride (TG) level for predicting 30-day and 1-year mortality in first-ever ischemic stroke

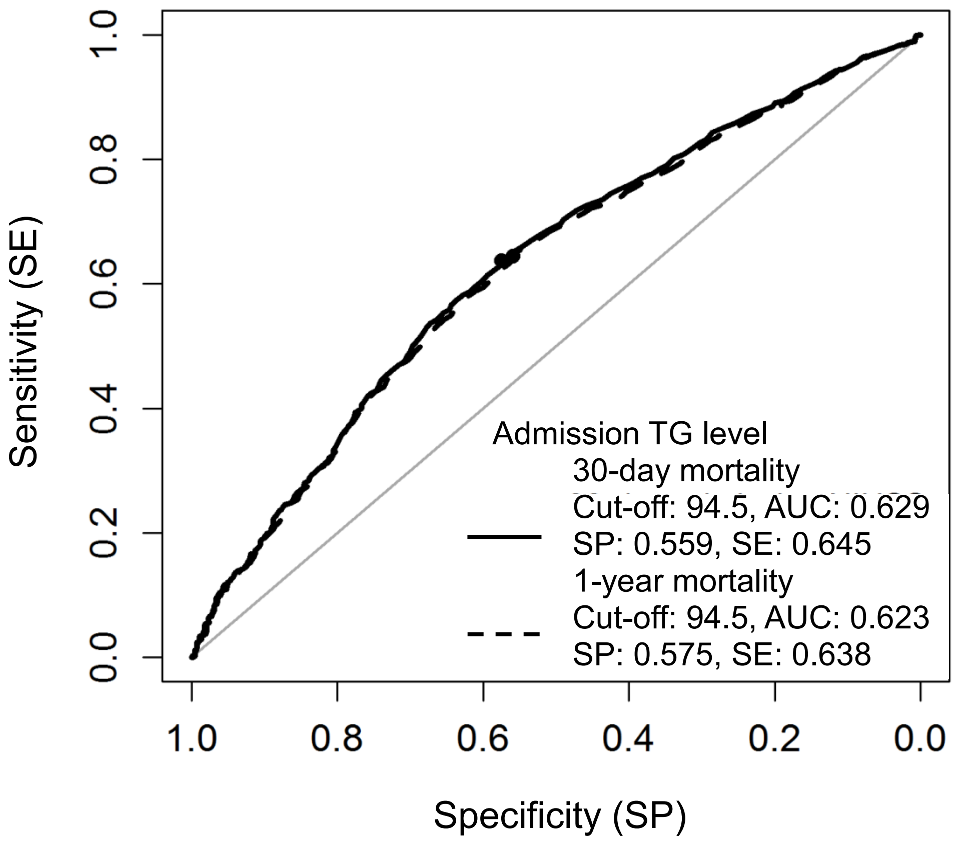

Receiver operating characteristic (ROC) curves to identify the cut-off points of admission non-high-density lipoprotein cholesterol (Non-HDL-C) level for predicting 30-day and 1-year mortality in first-ever ischemic stroke

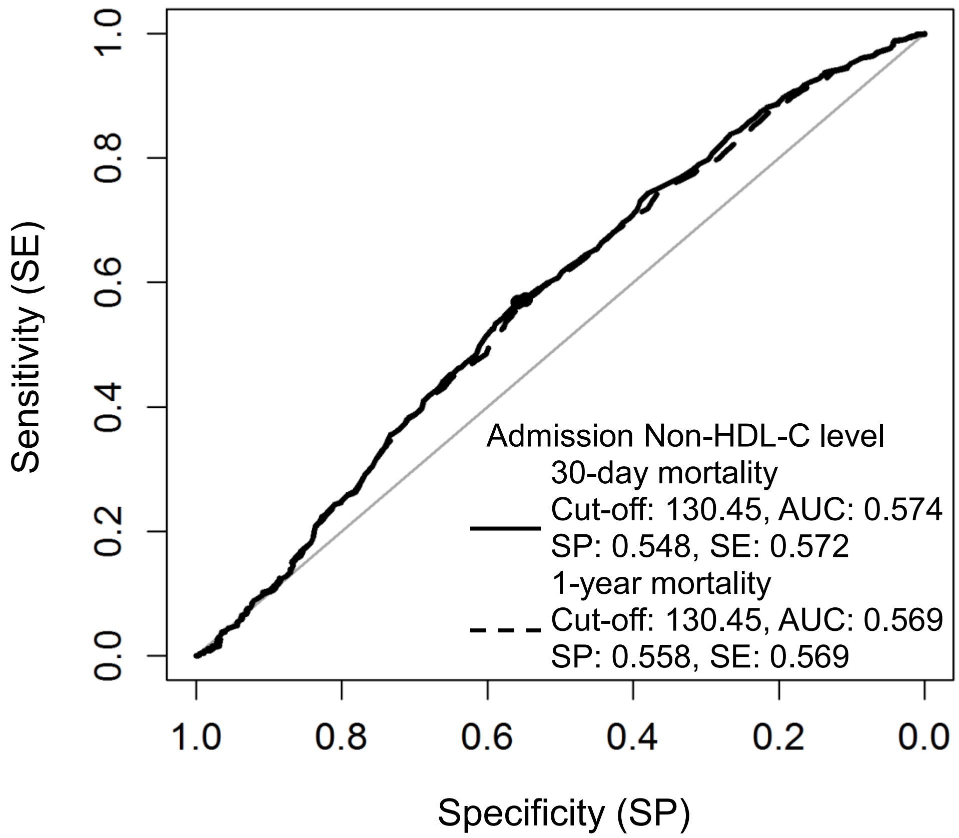

Receiver operating characteristic (ROC) curves to identify the cut-off points of admission high-density lipoprotein (HDL) level for predicting 30-day and 1-year mortality in first-ever ischemic stroke

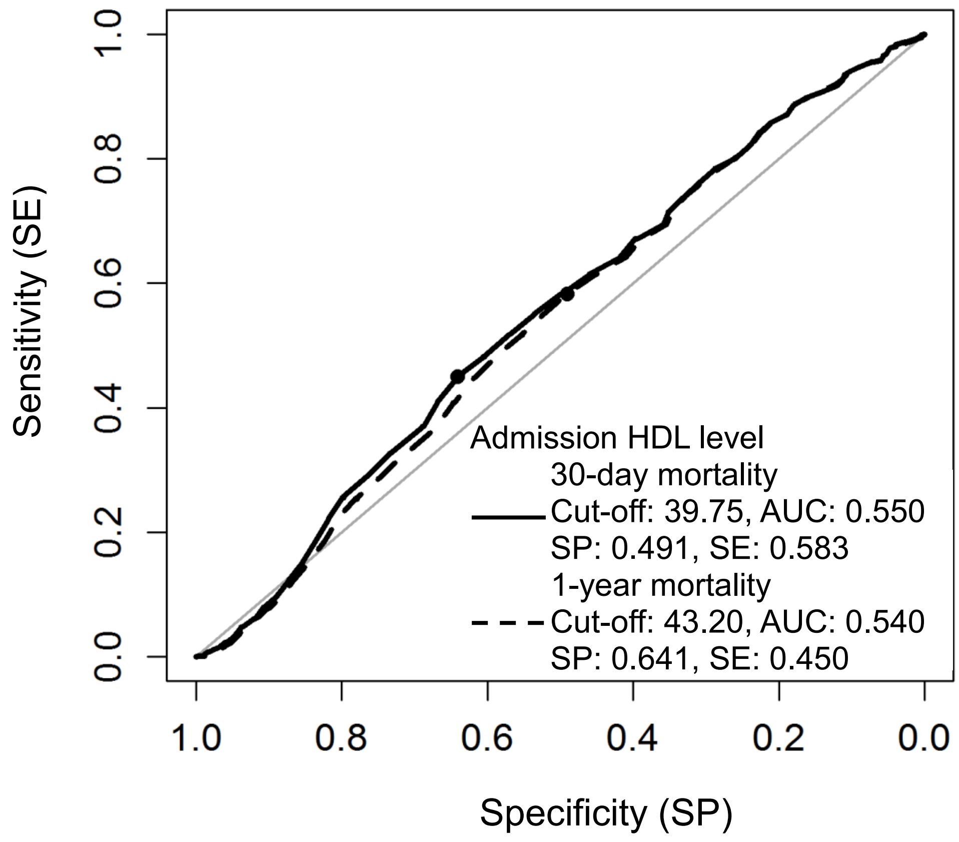

Receiver operating characteristic (ROC) curves to identify the cut-off points of admission total cholesterol/high-density lipoprotein (TC/HDL) ratio for predicting 30-day and 1-year mortality in first-ever ischemic stroke

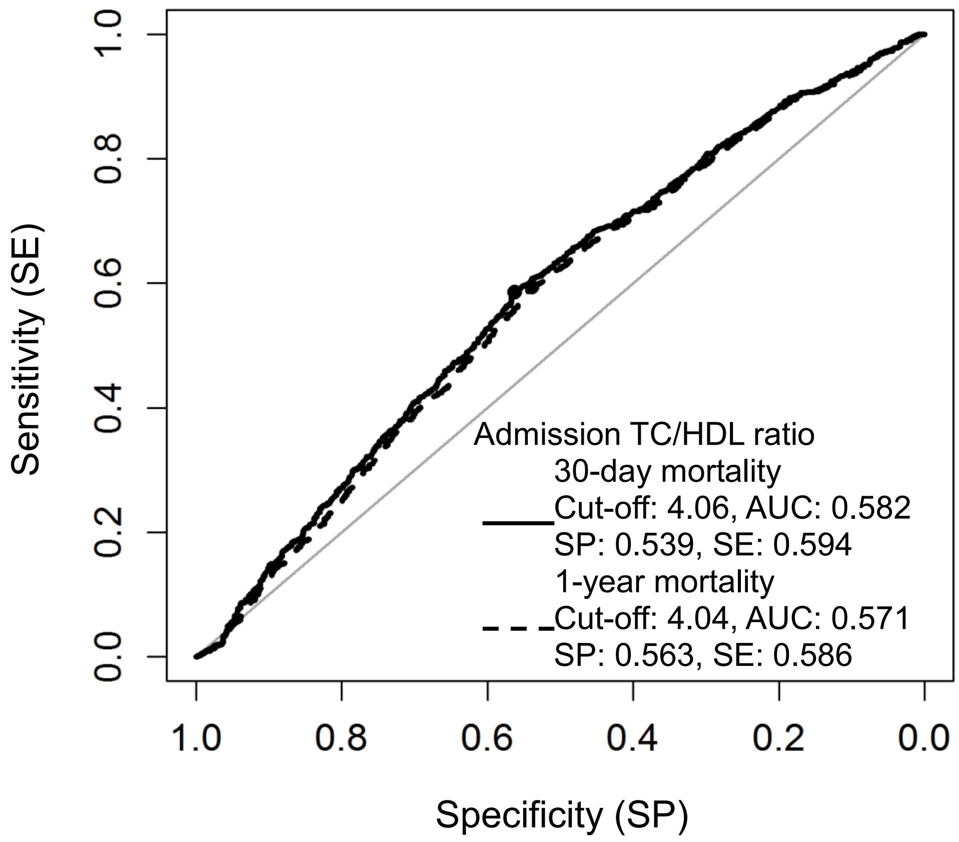

Receiver operating characteristic (ROC) curves to identify the cut-off points of admission diastolic blood pressure (BP) level for predicting 30-day and 1-year mortality in first-ever ischemic stroke

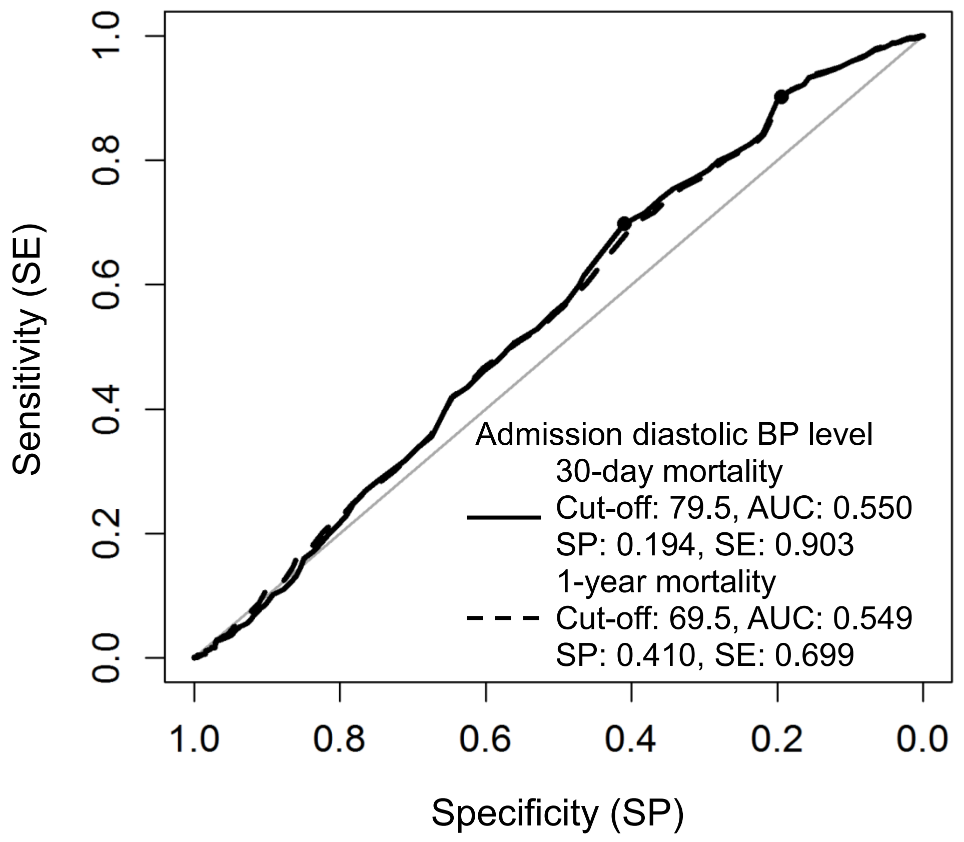

Receiver operating characteristic (ROC) curves to identify the cut-off points of admission mean blood pressure (BP) level for predicting 30-day and 1-year mortality in first-ever ischemic stroke

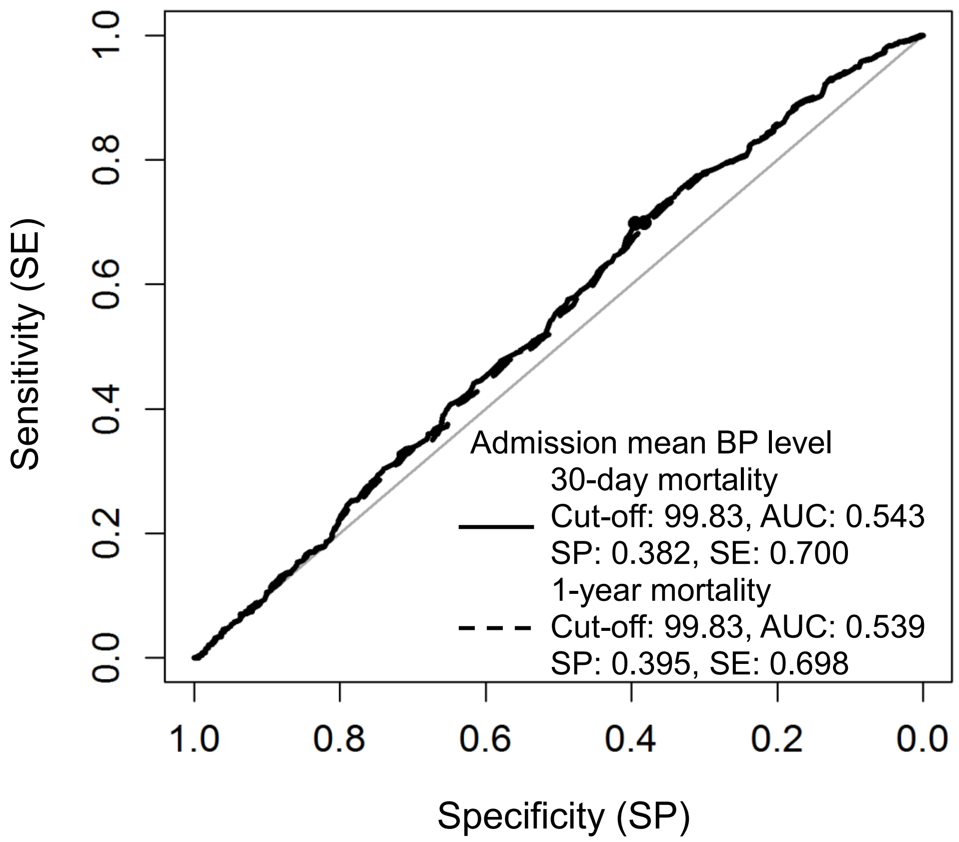

Receiver operating characteristic (ROC) curves to identify the cut-off points of admission fasting glucose (FG) level for predicting 30-day and 1-year mortality in first-ever ischemic stroke

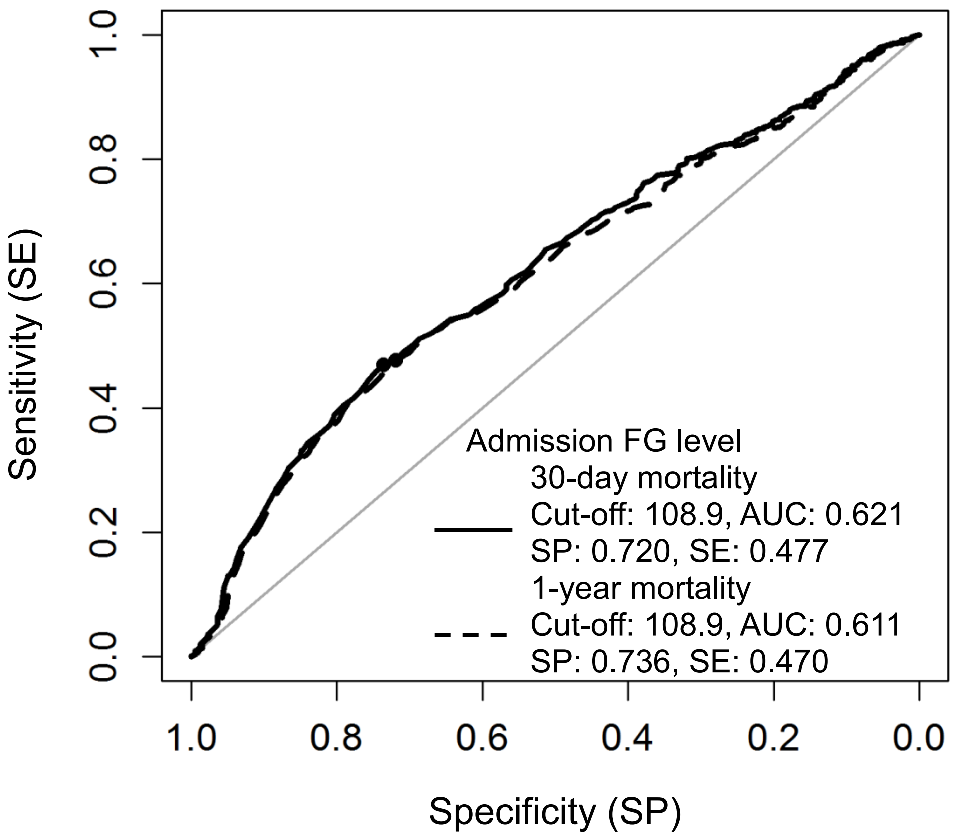

Hemorrhagic stroke

Receiver operating characteristic (ROC) curves to identify the cut-off points of admission NIHSS score for predicting 30-day and 1-year mortality in first-ever hemorrhagic stroke

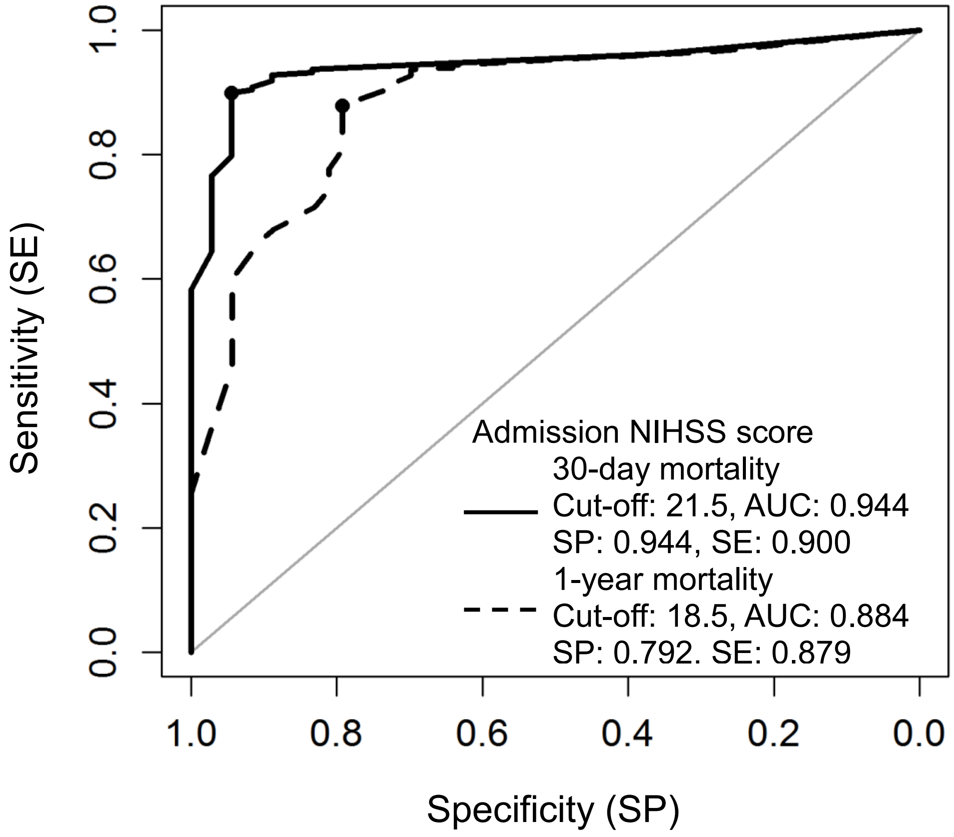

Receiver operating characteristic (ROC) curves to identify the cut-off points of admission diastolic blood pressure (BP) level for predicting 30-day and 1-year mortality in first-ever hemorrhagic stroke

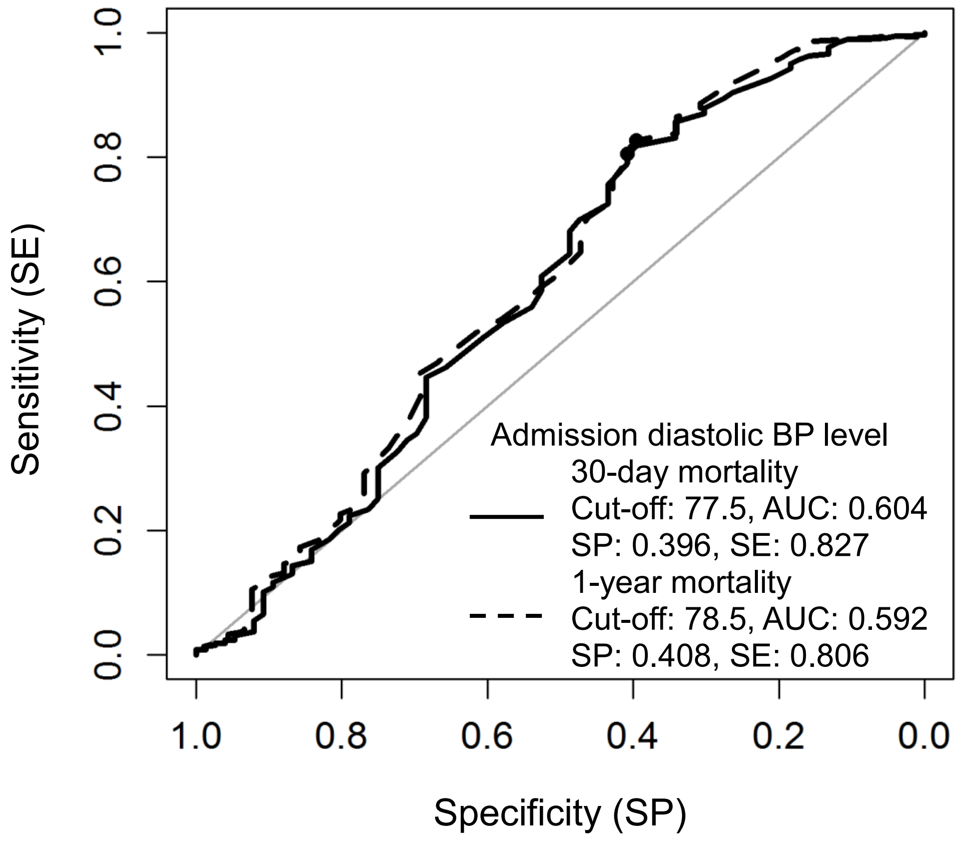

Receiver operating characteristic (ROC) curves to identify the cut-off points of admission fasting glucose (FG) level for predicting 30-day and 1-year mortality in first-ever hemorrhagic stroke

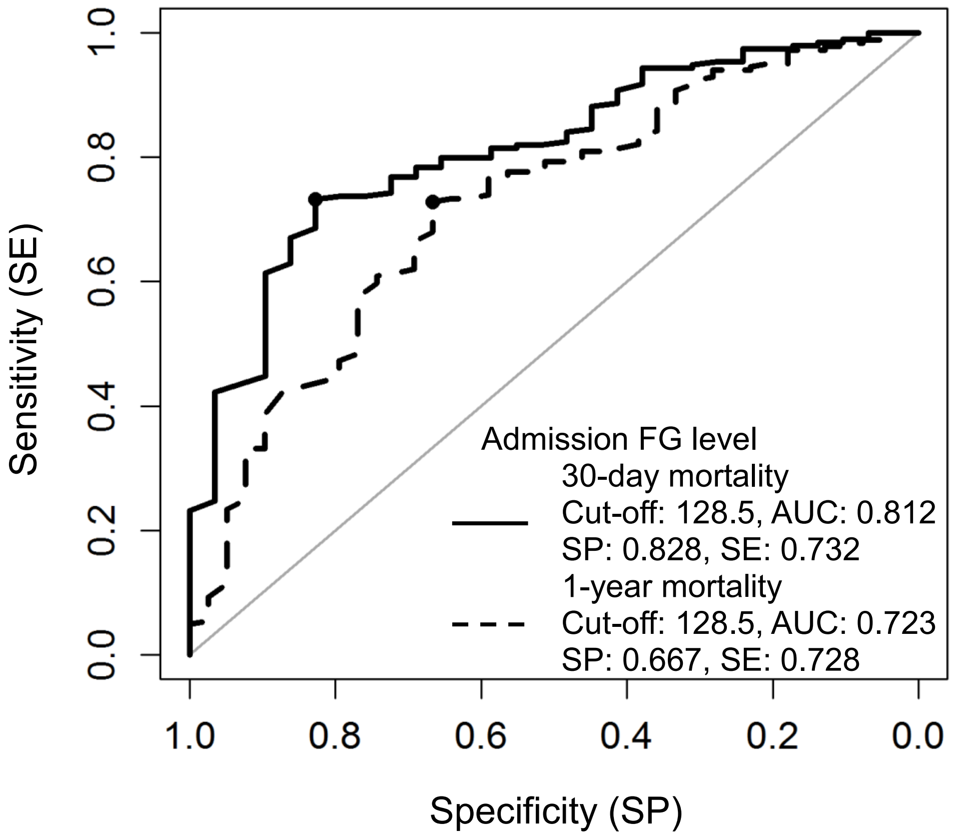

Supplement: Supplementary file 2 [file Presentation_1.PDF]
